# Intratumoral Microorganisms in Tumors: Current Understanding and Emerging Therapeutic Strategies

**DOI:** 10.1002/mco2.70754

**Published:** 2026-05-05

**Authors:** Haoling Zhang, Zengkan Du, Ping Lu, Aimin Jiang, Yadong Guo, Yawei Liu, Zhijing Song, Bing Dai, Wangzheqi Zhang

**Affiliations:** ^1^ Kidney Institute of CPLA, Department of Nephrology, Changzheng Hospital Navy Medical University Shanghai China; ^2^ Department of Biomedical Science, Advanced Medical and Dental Institute Universiti Sains Malaysia Penang Malaysia; ^3^ The First Affiliated Hospital of Henan Medical University Weihui Henan Province China; ^4^ Changhai Hospital Naval Medical University Shanghai China; ^5^ Department of Urology Changhai Hospital Naval Medical University (Second Military Medical University) Shanghai China; ^6^ Department of Urology The First Naval Hospital of Southern Theater Command Zhanjiang China; ^7^ Department of Urology, Shanghai Tenth People's Hospital Tongji University Shanghai China; ^8^ Naval Medical University Shanghai China; ^9^ Gansu University of Chinese Medicine Lanzhou Gansu Province China

**Keywords:** determinants of immunotherapy responsiveness, intratumoral microbiota, microbial regulation of antitumor immunity, microbiota‐derived metabolites, microbiome‐targeted therapeutic strategies, tumor microenvironment

## Abstract

Recent advances in high‐throughput sequencing, spatial omics, and integrative multiomics analyses have established reproducibly detectable microbial communities within tumor tissues, leading to the conceptualization of tumors as complex ecosystems encompassing an “intratumoral microbiota.” These microorganisms have increasingly been recognized as contributing to tumorigenesis, progression, and therapeutic response through interactions with the immune system, immunometabolic reprogramming of tissues, chronic inflammation, and genomic instability. Nevertheless, current evidence remains piecemeal and descriptive, with limited systematic consolidation of microbial composition, functional mechanisms, and translation to clinical application, particularly across tumor types and microenvironmental contexts. This review summarizes microbial diversity, tumor‐type‐specific associations, and multilayered mechanisms including immune modulation, metabolic reprogramming, and signaling rewiring, and discusses emerging applications such as biomarker discovery, prognostic stratification, and microbiome‐targeted therapeutics. Special focus is placed on tumor microenvironment, microbiota‐derived metabolites, and determinants of immunotherapy responsiveness. Overall, this review underscores the intratumoral microbiota as a dynamic and context‐dependent regulatory layer in cancer biology and offers an integrated framework to realize microbiome‐informed precision oncology, along with avenues for enhanced patient stratification and personalized therapeutic approaches.

## Graphical abstract

Tumors are no longer viewed as sterile but dynamic ecosystems harboring low‐biomass intratumoral microbiota. These microbes, derived from gut, oral cavity, or circulation, shape tumorigenesis and therapy response via core mechanisms: immunomodulation (regulating T cells, macrophages), metabolic reprogramming (SCFAs, indoles, bile acids), chronic inflammation, and genomic instability. Their composition and diversity serve as diagnostic/prognostic biomarkers. Emerging strategies—microbe‐based immunotherapies, engineered delivery systems (e.g., OMVs), and synergy with chemo/immunotherapy—target the microbiota to rewire TME. This microbiome‐informed framework enables precise tumor stratification and personalized oncology, bridging microbial ecology with cancer therapeutics.

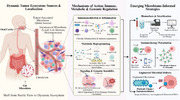

## 1 Introduction

Over the past decades, cancer has predominantly been regarded as a disease controlled by host‐cell genetic mutations, epigenetic alterations, and imbalance of the microenvironment with an emphasis on tumor intrinsic programs and interplay with the immune and stromal compartments. In this context, tumor tissue has been historically considered as a mainly sterile pathological site. The landscape has changed rapidly with the advent of high throughput sequencing, spatial transcriptomics, and integrative multiomics. A low‐biomass but transcriptionally active, and/or host‐state‐conditioned, microbial community is detected in a wide range of solid tumors and this presents as an offshoot of multiple studies as an intratumoral microbiota [1, 2]. This evidence reframes tumors as dynamic ecosystems comprising malignant cells, host immunity, and microbial residents, and adds an additional conceptual layer for understanding tumor evolution and therapeutic response.

In contrast to gut or mucosa‐associated microbiomes, intratumoral microbes are characterized by a very low microbial burden, strong spatial heterogeneity, and an intimate colocalization with host tissues. These bacteria are suggested to originate from the gut, oral cavity, or circulation and infiltrate tumors during initiation or progression, establishing residence and, in certain contexts, specialized niches [3, 4]. Despite being relatively few in number, their effects may be enhanced through localized interactions with tumor cells, immune infiltrates, and stromal elements [2]. At the same time, field‐wide reproducibility has been limited by the vast excess of host DNA, high risk of contamination, and methodological variability. These limitations highlight the need for strict experimental design and quality control, as well as a refocus from presence/absence to functional relevance.

Mechanistically, an emerging concept is that intratumoral microbes seldom function through one unmediated pathway of carcinogenesis; instead, their functional effects are more often mediated by modifying the tumor immune microenvironment. Microbial metabolites serve as immunoregulatory signals for immune‐cell metabolism, fate, and effector function reprogramming, while microbe‐associated molecular patterns activate innate sensors to promote local immune activation or tolerance to restore inflammation‐immunosuppression balance [5, 6]. In addition, some microbes may sustain low‐grade, persistent infection‐like states that perturb immune homeostasis, promote chronic inflammation, or alter cell‐death programs, facilitating immune evasion and adaptive tumor evolution. These intertwined mechanisms operate in a context‐dependent manner and likely contribute to intertumor and interpatient immune heterogeneity. However, much of the current evidence remains derived from associative analyses and preclinical models, and therefore causal relationships should be interpreted with caution.

With increased mechanistic clarity, the field is now progressing to clinical application. Increasingly, the composition and functional potential of intratumoral microbes are associated with prognosis, immunotherapy response, and treatment‐related toxicities in multiple tumor types, which is pointing to their development as predictive biomarkers or actionable modulators [7]. Engineered microbes, metabolite‐based interventions, microbiome remodeling, or combination with therapy are highlighting the preclinical potential in reprogramming local immunity to enhance antitumor response [8, 9]. In this article, “intratumoral microbiota” refers to microbial entities detected within the tumor tissue through either spatial localization or tissue‐level validation, while “tumor‐associated microbiota” denotes microbes in the gut, oral cavity, blood, and adjacent tissues that influence tumor biology indirectly via systemic or local effects. For conceptual clarity, this section centers mechanistic articles on intratumoral microbes and considers tumor‐associated communities predominantly as a systemic metabolic–immune landscape. Despite these advances in microbiome‐based therapeutic and translational research, important challenges remain, including marked tumor‐type specificity, limited safety evaluation, and the early stage of clinical translation [10, 11]. Therefore, this review summarizes recent progress in intratumoral microbiota research, with a focus on central mechanisms of immune regulation and emerging therapeutic approaches and future directions that may enable the generation of more accurate and controllable oncology‐based interventions.

## 2 The Intratumoral Microbiome

This section provides an overview of the intratumoral microbiome, focusing on its diversity, compositional characteristics, functional roles across microbial kingdoms, and tumor‐type‐specific associations.

### 2.1 Microbiome Diversity

With recent developments in metagenomic sequencing, 16S rRNA profiling and other single‐cell technologies, it is increasingly being recognized that tumor tissues are not consistently sterile but that many contain low‐biomass, albeit detectable microbial communities [12, 13]. Comprising primarily bacteria alongside detected viruses, fungi, and minor archaeal signals, these communities exhibit high compositional dissimilarity across tumor types, individuals, and spatial regions, highlighting the profound heterogeneity of the intratumoral microecology.

The majority of studies thus far have been descriptive in nature with the use of α‐ and β‐diversity measurements and reporting statistical associations with tumor type and response to treatment [14]. For instance, in NACI, variations in intratumor microbial diversity have been associated with response stratification, with patients achieving pathological complete response (pCR) displaying relatively higher α‐diversity and/or microbial burden [15]. Between tumors, diversity patterns frequently seemed to reflect systemic inputs or contributions from neighboring tissue that vary by tissue type: α‐diversity and β‐diversity differ significantly between tumor and peri‐tumor regions in hepatocellular carcinoma whereas in oral squamous cell carcinoma (OSCC), the dominant effect is a shift in β‐diversity compatible with community restructuring. Taken together, these observations indicate that tumor‐related changes in the microbiota more frequently reflect remodeling of community structure than mere variations in species richness [15, 16, 17, 18].

In line with this, intratumor microbial diversity is distinct among tumor types and response strata, patterns that are statistically associated with clinical outcomes and prognosis. More importantly, both α‐diversity and β‐diversity are capable of differentiating between pCR and non‐pCR outcomes [15, 19]. While mostly correlative rather than mechanistic, these data suggest that community‐level properties may be correlates of therapeutic sensitivity. This report presents global intratumor interface microbiota features observed from diversity settings, with functional implications and mechanistic deductions discussed in later sections.

### 2.2 Roles of Diverse Microorganisms

As the study of intratumoral microbiota is currently mostly focused on bacteria, and most mechanistic evidence also comes from research on bacteria, the functional impact of other microbial kingdoms remains relatively underexplored [20]. Intratumoral communities are, however, multikingdom consortia and an important constituent of the tumor microenvironment (TME). Tumor‐associated microbes can exert context‐dependent effects within the TME, ranging from promoting to suppressing tumor growth, thereby highlighting considerable functional heterogeneity [20, 21]. This diversity presumably mirrors variation in intratumoral spatial niche, host immune tone, local metabolic environment, and treatment history, which collectively govern crosstalk between microorganisms and hosts that links inflammation to immune control.

Within the current literature, tumor‐associated bacteria are the best characterized class, with several studies demonstrating enrichment in different tumor types and correlations with local inflammation/immune status. For instance, *Fusobacterium nucleatum* is associated with immuno‐evasion and responses to immunotherapy in multiple solid tumors; microbiota‐associated signals are statistically related to TREM2 deficiency and IL‐1β‐driven inflammation in pancreatic ductal adenocarcinoma (PDAC); the presence of *Helicobacter pylori* in tissues associated with gastric cancer (GC) correlates well with changes in epithelial phenotypes as well as expression of metabolism‐related molecules [22, 23, 24]. In contrast, despite the limited research on intratumoral fungi, these organisms have been identified in specific gastrointestinal tumor subgroups. To facilitate a systematic comparison of intratumoral microbes across origins, modes of regulation, and tumor‐biological associations, the key findings are summarized in Table 1. To date, there is an apparent enrichment of *Candida spp*. in digestive tract tumors and its association with inflammation, immunity, and metabolism‐related traits; however, results were not consistent across datasets indicating a marked context dependency [13, 25, 26]. Beyond bacteria and fungi, viruses—and, less frequently, archaea—have also been reported as components of intratumoral ecosystems, but their bona fide colonization and functional relevance require confirmation under stringent spatial validation and contamination control.

**TABLE 1:** Composition of the intratumoral microbiota and their functional roles in cancer.

Microorganism category	Representative microorganisms	Primary sources/routes of entry	Pathogenic/regulatory mechanisms	Key biological functions	Associated tumor types	References
Bacteria	*Fusobacterium nucleatum*	Commensal appendiceal microbiota, potentially via local invasion	Induces lymphocyte apoptosis and promotes M2 macrophage polarization	Negatively regulates T cells, positively correlates with macrophages, and is associated with improved survival	Appendiceal carcinoma	[30]
Bacteria	*Fusobacterium nucleatum* subsp. animalis	Oral origin with hematogenous dissemination	Disrupts cell–cell junctions and induces G0–G1 cell cycle arrest	Induces tumor cell dormancy and chemoresistance	Colorectal cancer (CRC) and oral squamous cell carcinoma (OSCC)	[31]
Bacteria	*Corynebacterium spp*., *Staphylococcus spp*.	Primarily derived from the nasopharyngeal microbiota	Interferes with cell cycle‐ and metastasis‐related pathways and suppresses antitumor immune responses	High microbial burden predicts poor prognosis and inhibits T‐cell infiltration	Nasopharyngeal carcinoma	[32]
Bacteria	Enterotoxigenic *Bacteroides* fragilis	Gut microbiota via intestinal colonization	Induces regulatory T‐cell differentiation	Promotes tumor progression through Treg induction	CRC	[33]
Bacteria	*Stenotrophomonas maltophilia*	Widely distributed in natural and healthcare environments	Activates the TLR4/nuclear factor kappa B (NF‐κB) pathway to induce inflammation	Promotes immunosuppression and shortens patient survival	Pancreatic ductal adenocarcinoma (PDAC)	[34]
Bacteria	*Streptococcus spp*.	Translocates to the breast following disruption of the intestinal barrier	Promotes inflammation and impairs intestinal barrier function	Facilitates breast tumor initiation and progression	Breast cancer (BC)	[35]
Fungi	*Malassezia spp*.	Intratumoral colonization	Downregulates CYP7A1 and related genes to suppress bile acid synthesis	Modulates the tumor microenvironment to promote hepatocellular carcinoma progression	Hepatocellular carcinoma	[36]
Bacteria	*Clostridium butyricum*	Intratumoral microbial colonization	Triggers oxidative stress and lipid accumulation, thereby enhancing ferroptosis	Enhances ferroptosis sensitivity and suppresses tumor progression	PDAC	[37]
Bacteria	Helicobacter cinaedi	Transcriptionally active intratumoral microbiota	Associated with SELENBP1 gene expression affecting survival	High expression correlates with poor survival outcomes	Metastatic CRC	[38]
Bacteria	*Lachnospiraceae spp*.	Derived from translocated gut microbiota	Induces inflammatory responses and promotes epithelial–mesenchymal transition (EMT) programs	Shapes immune phenotypes and influences responses to immunotherapy	Bladder urothelial carcinoma	[39]
Bacteria	*Fusobacterium nucleatum subsp*. animalis	Oral origin	Induces reactive oxygen species (ROS) production and DNA damage and activates NF‐κB signaling	Associated with poor prognosis and a proinflammatory microenvironment	Esophageal adenocarcinoma	[40]
Fungi	Alternaria alternata	Retrograde migration from the gut microbiota through the sphincter of Oddi	Activates the Dectin‐1 pathway and induces IL‐33 secretion	Recruits T helper 2 (TH2) and Type 2 innate lymphoid cells (ILC2) to promote tumor progression	PDAC	[41]
Fungi	*Malassezia globosa*	Skin‐resident commensal, potentially disseminated via hematogenous or direct routes	Activates the IL‐17A/macrophage axis and the TLR4/NF‐κB pathway	Accelerates tumor growth, promotes M2 polarization, and induces lipid dysregulation	BC	[42]
Bacteria	*Helicobacter pylori*	Gastric mucosa‐resident bacterium acquired via oral transmission	Induces chronic inflammation, remodels the tumor microenvironment, and triggers specific gene expression	Associated with poor prognosis, enhanced immune infiltration, and chemotherapy resistance	Gastric cancer (GC)	[43]
Fungi	*Candida albicans*	Oral or gastrointestinal commensal microbiota	May promote carcinogenesis by reducing fungal diversity	Serves as a biomarker for GC and may contribute to tumorigenesis	GC	[44]
Bacteria	*Sphingomonas paucimobilis*	Derived from environmental sources or translocated gut microbiota	Promotes CCL20 secretion to recruit regulatory T cells	Suppresses tumor immune surveillance and promotes BC progression	Triple‐negative BC	[45]
Fungi	*Malassezia spp*.	Migrates from the gut to the pancreas	Activates the MBL‐complement cascade	Promotes pancreatic cancer initiation and progression	PDAC	[46]
Bacteria	*Fusobacterium nucleatum*	Intratumoral bacterial invasion of melanoma cells	Bacterial peptides are presented via HLA molecules to activate T‐cell immunity	Modulates the tumor immune microenvironment and influences therapeutic responses	Melanoma	[47]
Bacteria	*Streptococcus spp*.	Normal microbiota of the oral and head‐and‐neck mucosa	Produces acetaldehyde, which may promote oral carcinogenesis	Altered abundance correlates with tumor T stage	Oral tongue squamous cell carcinoma	[48]
Fungi	*Cladosporium cladosporioides*	Environmental exposure via oral entry	Induces tissue injury, inflammation, and DNA damage	Drives the development of esophageal squamous cell carcinoma	Esophageal squamous cell carcinoma	[49]
Virus	HHV‐6	Latent infection reactivation or direct infection of tumor cells	Positively correlates with NK‐cell infiltration	Drives NK‐cell infiltration and is associated with improved prognosis	Soft tissue sarcoma	[50]
Bacteria and fungi	*Capnocytophaga spp*., *Haemophilus parainfluenzae*	Local invasion from the oral cavity or adjacent tissues	Modulates oncogenic and metastasis‐associated pathways	Associated with carcinogenic, metastatic, and inflammatory pathways and impacts prognosis	Head and neck squamous cell carcinoma	[51]

Abbreviations: BC, breast cancer; CRC, colorectal cancer; EMT, epithelial–mesenchymal transition; GC, gastric cancer; ILC2, Type 2 innate lymphoid cells; NF‐κB, nuclear factor kappa B; OSCC, oral squamous cell carcinoma; PDAC, pancreatic ductal adenocarcinoma; ROS, reactive oxygen species; TH2, T helper 2.

Microbiota‐derived from different origins and with diverse functional modes of action converge on core intratumoral processes—such as inflammation, immune escape, and metabolic reprogramming—processes that codetermine the multifaceted and dynamic tumor meta‐ecosystem [27, 28]. This microbe‐modulated regulatory circuitry may influence tumorigenesis and may also contribute to therapeutic response, thereby providing a conceptual foundation for mechanism‐based, microbiome‐informed precision interventions, and the rational design of novel treatments [10, 29].

### 2.3 Microbiome–Tumor Type Associations

In the past few years, with accumulating evidence from high‐throughput sequencing and metagenomics, it has become clear that microbial communities can be identified within tumor tissues, as well as within both intratumor and tumor‐associated microbes that differ strikingly across cancer types [52]. These findings are inconsistent with a random dispersal of microbes, but suggest tumor‐type‐related and tissue‐specific patterning patterned by observable biological laws. Crucially, different tissues present their own set of ecological constraints and immunological barriers. Therefore, tumor‐associated microbial signatures might reflect selective enrichment driven by the local environment of the stroma, but could also be influenced indirectly from distant mucosae/organs and even by host systemic metabolic–immune status, in relation to microbes. The latter should not be confused with real intratumor colonization, as the burden of proof and spatial attribution are different, yet such distal influences may still remodel the tumor niche, which in turn can influence local microbial composition and function.

In gastrointestinal malignancies, the tumor‐associated microbiota shifts are defined by simultaneous metabolic dysregulation and impaired inflammatory/immune control. Several studies have also generated similar findings that beneficial taxa related to short‐chain fatty acid (SCFA) production and mucosal homeostasis were depleted in GC and CRC, whereas proinflammatory and protumorigenic organisms are relatively enriched [53, 54, 55]. These changes are associated with the local metabolic conditions and inflammatory tone and mucosal immune traits. Similar tumor‐type correlations are observed outside the GI tract. For example, lung cancer has been correlated with increased airway bacterial load and lower microbial diversity similar to the co‐occurrence of local dysbiosis and tumor‐associated inflammation [56]. In liver cancer, the emergence and progression of NASH–HCC has been closely linked to gut dysbiosis, underscoring the gut–liver axis as a potential conduit through which metabolic derangement and inflammatory signaling may contribute to hepatocarcinogenesis [57].

Apart from tissue context, diet and systemic metabolic status may remodel microbial community motifs with wide‐ranging effects across tumor types likely by changing the immunometabolic set points and basal inflammatory tone that secondarily reprogram tumor ecological niches. In the context of a high‐fat diet, microbiome perturbation has been reported to be associated with enhanced tumor growth in breast cancer, melanoma, and prostate cancer models, potentially via metabolic reprogramming and immune regulatory mechanisms, while microbiome‐targeted interventions may limit tumor progression, at least at the transcriptomic level [58, 59]. In the same way, the gut microbiota has also been associated with glioblastoma risk, progression, and response to therapy via immunometabolic pathways [60].

**FIGURE 1:**
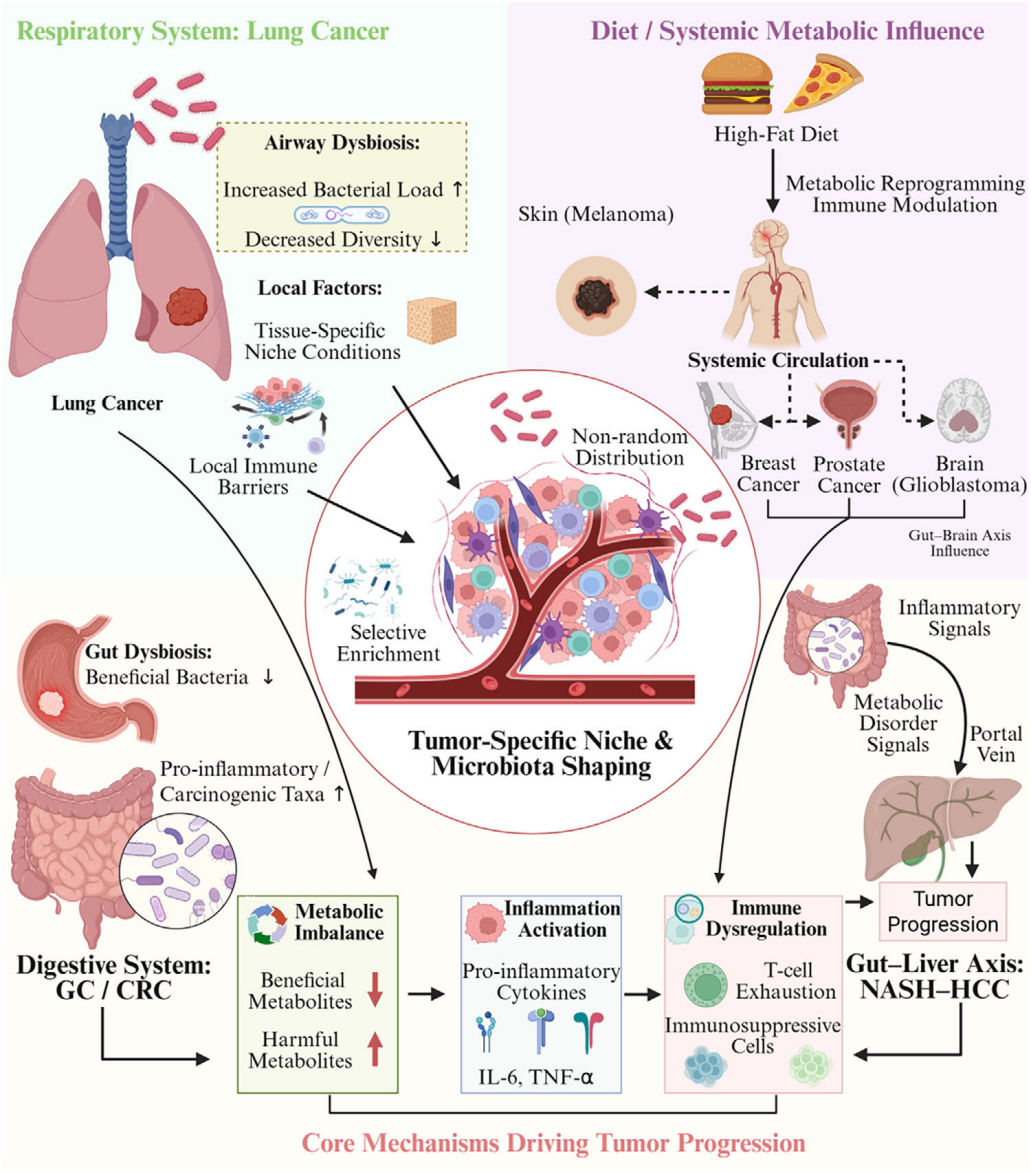
Tumor‐type‐specific associations of the intratumoral microbiome. The intratumoral microbiome is shaped by tissue ecology and host‐selective forces, leading to a nonrandom distribution that is partly tumor‐type specific. In lung cancer, airway dysbiosis (overconsumption rather than interaction) features elevated bacterial density and lower community diversity to impair local immune barrier and allow the accumulation of reactive oxygen species (ROS)/reactive nitrogen species (RNS)/oxidative DNA lesions; stress is exacerbated by compromise of mismatch repair (MMR) and nucleotide excision repair (NER) system, thus undermining genome integrity. In gastrointestinal cancers, gastric cancer (GC)‐associated and colorectal cancer (CRC)‐associated dysbiosis specifically promotes the outgrowth of proinflammatory/tumorigenic microorganisms. Short‐chain fatty acids (SCFAs) and the microbial metabolites trimethylamine N‐oxide (TMAO) and the secondary bile acid DCA may reach systemic circulation to influence distal tumor sites. Persistent sensing of microbial signals by tumor‐infiltrating myeloid cells activates calcineurin–nuclear factor of activated T cells (NFAT) and nuclear factor kappa B (NF‐κB) signaling, leading to chronic IL‐6, IL‐1β, and CXCL8 release that creates a proinflammatory microenvironment that can suppress immunity and increases expression of immune‐evasion checkpoints (PD‐L1, B7‐H3, and B7‐H4), which promotes CD8^+^ T‐cell exhaustion and dampens effector function. Together, these models portray the microbiome as a context‐dependent evolutionary amplifier that promotes tumor initiation and malignant progression but whose causal potency is limited by both host genetics and microenvironmental state (by BioRender). CRC, colorectal cancer; GC, gastric cancer; MMR, mismatch repair; NFAT, nuclear factor of activated T cells; NF‐κB, nuclear factor kappa B; NER, nucleotide excision repair; RNS, reactive nitrogen species; ROS, reactive oxygen species; SCFA, short‐chain fatty acid; TMAO, trimethylamine N‐oxide.

In summary, cancers display generally specific, but not unique, sets of microbiome alterations that likely reflect differential readouts of tissue‐mediated ecology in conjunction with systemic metabolic–immune forces. Numerous outward variances coalesce around core dysfunctions, including metabolic disruption, inflammatory excitation, and immune deviation, manifested in a tissue‐dependent manner. Mapping such tumor type–microbiome–mechanism relationships clarifies the concept of microenvironmental heterogeneity as a whole and offers insight into how these relationships can be leveraged for microbiome‐based tumor stratification, risk assessment, and precision therapy development. As illustrated in Figure 1, intratumor microbiota establish nonrandom, tumor‐type‐specific regulatory networks under tissue ecology, and host selection pressures, thereby providing an upstream ecological substrate for inflammation signaling and defective DNA repair.

## 3 Microbial Interactions Within the TME

This section provides an overview of the key modes of interaction between intratumoral microorganisms and the TME, with a focus on immune modulation, metabolic reprogramming, and direct microbe–tumor communication.

### 3.1 Interactions With the Host Immune System

Immune tone of the TME is generally considered a determining factor in tumor initiation, development, and therapeutic response. As more evidence comes to hand, the long‐standing dogma that tumors are “relatively sterile” has been reconsidered. Microorganisms within tumors can no longer be regarded as merely innocent bystanders, but are increasingly seen as potential partners that shape the immune landscape [27]. Through provision of variably local immunostimulatory signals, microbial cues may be integrated into innate and adaptive control networks, potentially contributing to tumor‐specific and stage‐specific immune states. This section is concerned with the immediate impact of intratumoral micro‐organisms on the tumor immune environment, achieved by influencing immune‐cell function and spatial localization. It should be noted that much of the evidence discussed in this section is derived from preclinical models, spatial colocalization analyses, or associative studies, and therefore the causal roles of intratumoral microorganisms remain to be further validated in a tumor‐type‐ and context‐specific manner.

At the level of innate immunity, associations between intratumoral microorganisms and immune responses are best understood in terms of their effects on myeloid differentiation, functional programs, and tissue localization opportunities. Moreover, microbe‐associated signaling has been associated with alternative monocyte fates to inflammatory states or immune‐suppressive tumor‐associated macrophages (TAMs) that subsequently alter the capacity for antigen presentation and local immune responses [2, 61, 62]. Further, microorganisms are nonuniformly distributed throughout the tumor tissue and frequently colocalize with areas within the tumor that are enriched in myeloid cells but poor in effector T cells, suggesting a link between microbial geography and immune‐suppressive architecture [2]. Of interest, the spatial coupling in these systems might be two way: immunosuppressive niches could locally allow for selective microbial engraftment and conversely microbial persistence may amplify local suppression.

Direct modulation of T cell‐mediated adaptive immune responses is mostly indirect, as microorganisms affect the maturation of dendritic cells and their efficiency in presenting antigen. Under specific circumstances, some microbial signals have been reported to promote the activation and expansion of intratumoral CD8^+^ T cells, which may contribute to cytotoxic immunity and potentially limit tumor growth [62]. Nevertheless, these effects are not universal across all tumors, manifesting instead in a context‐dependent manner. Certain models of microbe‐elicited immunity shift the balance toward protumorigenic activity. For instance, in the tumor context of pancreatic cancer, intratumoral fungi have been shown in specific models to induce IL‐33 expression by tumor cells and subsequently recruit and activate T helper 2 (TH2) cells and Type 2 innate lymphoid cells (ILC2s) leading to a Type 2‐biased microenvironment that promotes disease [41]. *Microorganism*s may also enhance regulatory T cell recruitment and function, sustaining local tolerance; this axis has been implicated in CRC development and progression [33].

**FIGURE 2:**
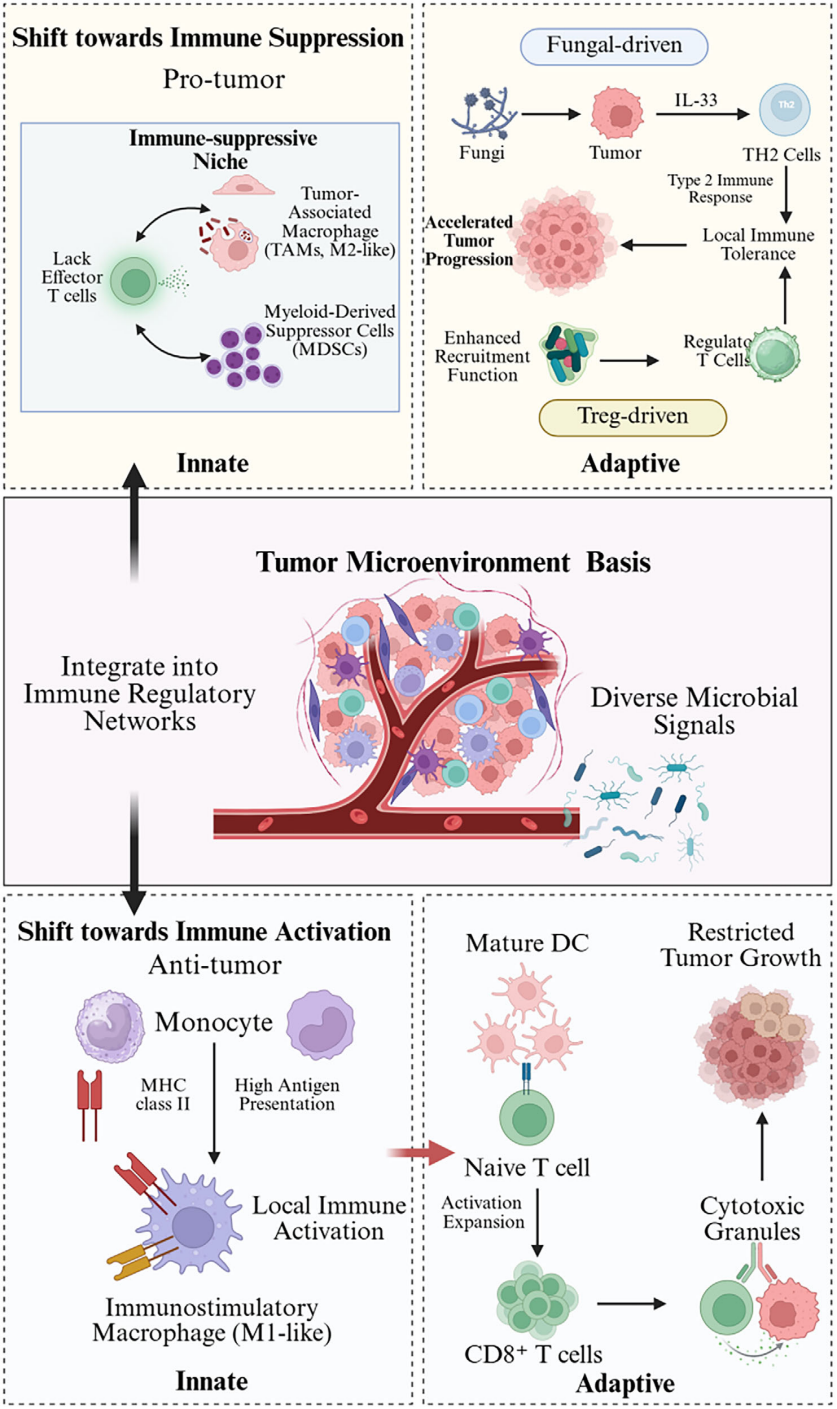
Intratumoral microbe‐driven immunoregulatory circuitry in the tumor microenvironment (TME). Tumor‐intrinsic microbiota mold a profoundly flexible immunoregulatory network in the TME, fomenting an ever‐changing battle between antitumor activation and protumor tolerance. There are two major pathways of fungal‐mediated regulation. Primarily, a fungal inflammatory tolerance axis where tumor‐cell IL‐33 release is provoked by fungal stimuli, resulting in T helper 2 (TH2)‐based immune priming and the establishment of an ectopic tolerogenic environment that potentiates tumor growth. Second, a Treg‐mediated immunosuppressive loop initiated by chronic microbial sensing of myeloid cells through the calcineurin–nuclear factor of activated T cells (NFAT)/nuclear factor kappa B (NF‐κB) pathway that sustains its activation and consequently contributes to constant IL‐6, IL‐1β, and CXCL8 production as well as expression of checkpoint molecules (for example, B7‐H3 and B7‐H4). This supports Treg recruitment and stability, leading to CD8^+^ T cell exhaustion and dysfunctional effector function. In contrast, specific microbes induce monocyte‐to‐dendritic‐cell maturation and a high antigen presentation state that supports naïve T cell priming/expansion and GrzB‐associated CD8^+^ cytotoxic degranulation, which controls tumor growth. Together, the microbiota function as an upstream ecological layer to shape the immune hierarchy and functional output of the TME; the global readout is codetermined by microenvironmental context and host responsiveness (by BioRender). NFAT, nuclear factor of activated T cells; NF‐κB, nuclear factor kappa B; TH2, T helper 2; TME, tumor microenvironment.

Cumulatively, intratumoral microbiota contribute to the balance of innate and adaptive immunity as a highly flexible regulatory interface [63]. Microbial influences, however, do not act as linear fixed effects; instead, microbial effects tend to skew toward immune activation or suppression on the basis of tumor type, community composition, and host immune state, which sets the stage for later remodeling of metabolic pathways and tumor‐cell signaling. Contradictory results among studies are almost certainly due to the inherent heterogeneity of the TME and highlight the importance of context‐resolved, systems‐level dissection of microbe–immune interactions. Understanding these conditional regulatory modes will be critical to precision implementation of microbiome‐mediated immunomodulatory approaches. As illustrated in Figure 2, intratumoral microorganisms cross talk with myeloid and lymphoid cell populations to shape either immunosuppressive or immune‐activating ecologies, with directionality and amplitude dictated by the dynamic balance between TME context and host immunity. Together, these findings highlight the multifaceted and context‐dependent roles of intratumoral microbiota in shaping immune, metabolic, and signaling networks within the TME [28].

### 3.2 Microbial Modulation of Tumor Metabolism

The metabolic reprogramming is one of the characteristics of TME. It is not exclusively a cell‐autonomous result of cancerous metabolic dysfunctions, but rather an important systemic phenotype codetermined by tumor cells together with the immune system and a variety of environmental signals. Emerging evidence suggests that intratumoral microbes may shape local metabolic profiles directly through the catalysis of substrate conversions, modulation of metabolic flux, and propagation of metabolic signals, whereas tumor‐associated microbes are primarily implicated in remodeling systemic metabolism and immunological states to affect cancer cell metabolism indirectly. This section will concentrate on how microbes may reshape tumor metabolic pathways and immunometabolic tone by direct or indirect ways.

At the molecular scale, microbe‐derived metabolites may function as nodal signals to reshape local metabolic axes and, through metabolism–immune coupling, influence antitumor immunity [64]. For instance, microbial indole metabolites have been reported to inhibit the production of critical enzymes in the tryptophan catabolic pathway and reduce immunosuppressive intermediate accumulation, thus modulating the host TME immunometabolic balance [65]. Spatially resolved metabolomics provides further evidence for a link between microbial colonization and regional metabolic phenotypes: niches that are colonized by colibactin‐producing *Escherichia coli* show an accumulation of glycerophospholipids, suggesting coordinated spatial coupling between the distribution of microbes and local lipid remodeling [66]. Furthermore, SCFAs have been reported to promote cytolytic T‐cell function in defined experimental settings through metabolic and epigenetic reprogramming in a defined concentration and immune context, highlighting the potential cooperative effects of microbial metabolism on immunometabolic regulation [67]. On a systemic level, the gut microbiota—a key part of tumor‐associated microbial communities—may modulate amino‐acid availability, glucose utilization, and cellular bioenergetics, thus contributing to tumor‐mediated metabolic rewiring that supports growth and immune evasion [68].

Importantly, microbe‐regulated metabolism is profoundly context dependent and not universally proimmune. In certain tumor types and metabolic conditions, SCFAs inhibit histone deacetylases, induce chromatin decompaction, and increase DNA damage, potentially promoting genomic instability and progression [69]. Dysbiosis in bile‐acid metabolism is another axis of microbial influence on tumor metabolic rewiring. Disturbed microbial conversion of bile acids can alter tumor metabolic status, and provides a link to therapeutic manipulation of secondary bile acids in cancer cachexia via modulation of hepatic cholesterol homeostasis; on the other hand, intestinal‐generated primary bile acids activate MAPK signaling and expression of oncogenic gene programs—highlighting the two‐way connective road between microbe and tumor metabolism [70, 71].

**FIGURE 3:**
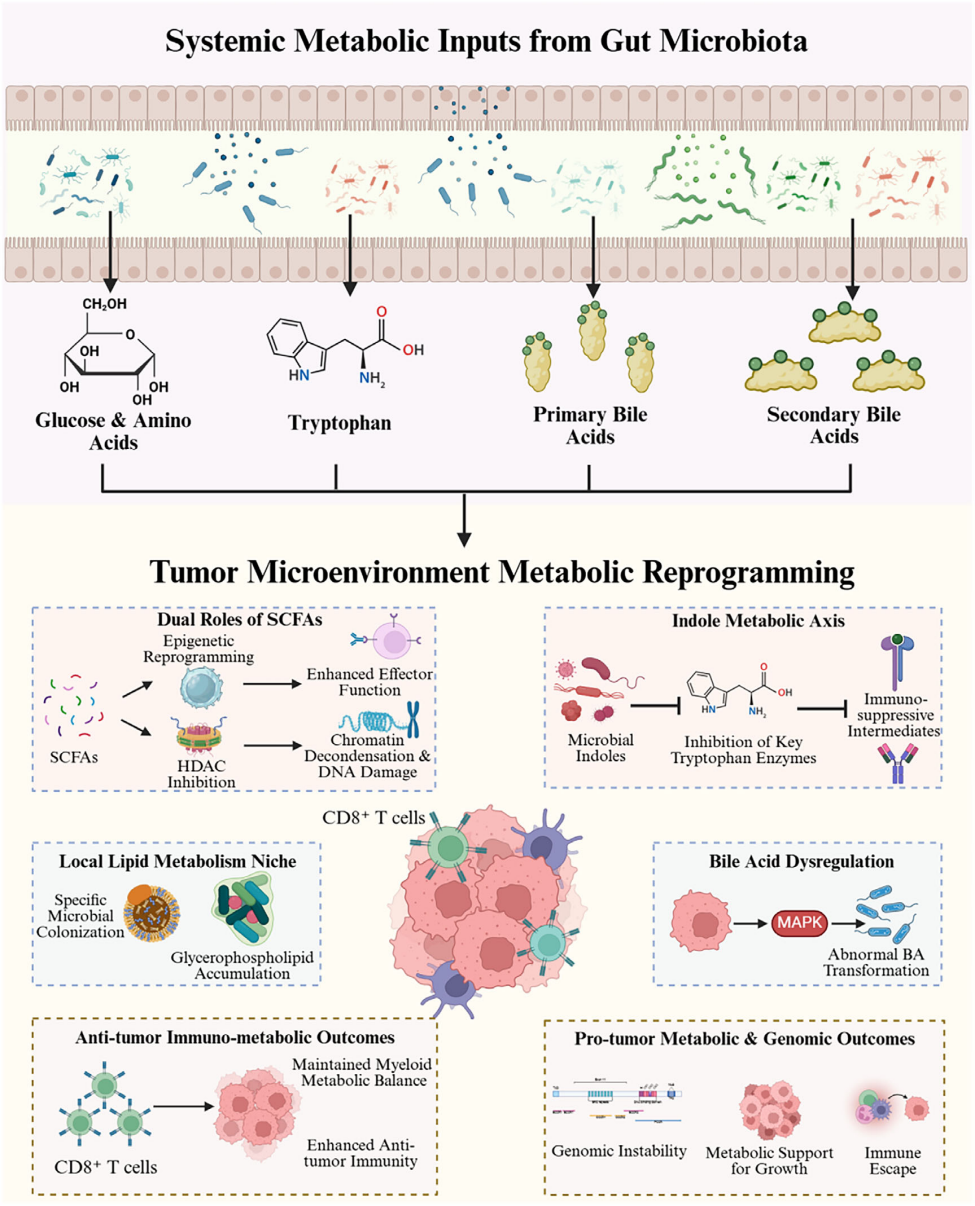
Microbiome‐associated metabolic reprogramming in the tumor microenvironment (TME). Coordinated actions of gut microbiota‐derived systemic metabolic fluxes and the local ecology of the TME underlie the regulation of metabolic reprogramming. At the nutrient supply level, glucose as well as amino acids (tryptophan included) and primary and secondary bile acids are predominant circulating substrates; microbe‐encoded signals enable the translocation of metabolites across barrier tissues into circulation that helps define metabolic inputs at tumor sites. Short‐chain fatty acid (SCFAs) exert bidirectional immunometabolic effects: by inhibiting histone deacetylases (HDACs), these metabolites relax chromatin and promote epigenetic remodeling, enhancing CD8^+^ T‐cell effector programs; yet, in the context of local lipid disequilibrium, these molecules may cooperate to trigger chromatin decompaction and DNA damage. Within the indole–tryptophan axis, microbial cues suppress key tryptophan‐catabolic enzymes [indoleamine 2,3‐dioxygenase (IDO)/tryptophan 2,3‐dioxygenase (TDO)], generating immunosuppressive intermediates and reinforcing a tolerance‐permissive niche. Bile‐acid imbalance can engage mitogen‐activated protein kinase (MAPK)‐dependent aberrant transformation signaling and, together with lipid‐tropic colonization, drive glycerophospholipid accumulation—establishing a metabolic milieu that supports tumor growth and immune escape. Collectively, these interactions position the microbiota as a context‐dependent upstream layer of metabolic control that can amplify coupled circuits of metabolic imbalance and immune exhaustion, promoting mutational accrual, therapy resistance and malignant progression, with net outcomes determined by the metabolic state of the TME and the caliber of host immunity (by BioRender). HDACs, histone deacetylases; IDO, indoleamine 2,3‐dioxygenase; MAPK, mitogen‐activated protein kinase; SCFA, short‐chain fatty acid; TDO, tryptophan 2,3‐dioxygenase; TME, tumor microenvironment.

In aggregate, intratumoral microorganisms exert a malleable immunoregulatory stratum by shaping the balance between innate and adaptive immunity, whose outcomes depend on tumor type and microenvironmental setting. Concurrently, intratumoral and tumor‐associated bacteria modify core pathways of carbon, amino‐acid, lipid, and bile acid metabolism, linking metabolic reprogramming to immune regulation, inflammatory tone, and genome stability in the establishment of a site‐specific metabolic niche. Together, these observations suggest that tumor metabolism is not determined solely by cancer cell‐intrinsic properties, but continuously negotiated via microbe‐host interactions—a framework that could serve to understand the evolution of tumors through a metabolic lens and to design microbiome‐based therapeutic interventions. Nevertheless, much of this mechanistic interpretation remains inferential, and the relative contribution of microbes to metabolic rewiring is likely to vary substantially across tumor contexts and experimental systems [8, 9]. Figure 3 illustrates how microbe‐driven nutrient‐flux remodeling and imbalances in SCFA, indole, and bile‐acid signaling intersect as upstream triggers of TME metabolic reprogramming, further reinforced by epigenetic programming and immune effector circuits, thereby enhancing selective pressures during tumor evolution.

### 3.3 Microbiota–Tumor Communication

Further to this indirect influence on tumor progression via immune and metabolic effects, intratumoral microbes may communicate with and alter the function of tumor cells through multiple layers of dynamically adjustable crosstalk, which connects with both signaling circuitry and stress‐response pathways to modify proliferative potential, phenotypic stability, and susceptibility to therapy. There is growing evidence that these microorganisms are not merely passive responders of the microenvironment, but instead, via structural components, metabolites, and soluble mediators engage in direct molecular interactions that may modulate tumor‐cell signaling states and behavior.

At the level of interkingdom signaling, microbial structural ligands and their resultant signals represent a prototypical mechanism of direct microbe–tumor cell interaction. Thus, for example, the lipopolysaccharide (LPS)–TLR4 axis has been proposed to relay into and trigger an NF‐κB–IL‐6–STAT3 signaling module in tumor cells that may support proliferative, inflammatory oncogenic programs, as well as treatment‐resistant phenotypes. Pharmacological targeting of this axis also strongly suppresses initiation and progression of inflammation‐driven tumors in line with a cell‐extrinsic protumor role in various models [72, 73]. More directly, selected taxa enriched within tumor tissue and their metabolites may function as functional signaling units that intersect with canonical oncogenic pathways. Downstream of the Wnt/β‐catenin pathway, *Fusobacterium mortiferum* and its metabolite 5‐aminovaleric acid have been observed in specific experimental systems to inhibit the tumor suppressor DKK2 and activate Wnt/β‐catenin signaling, thus creating a line of direct molecular crosstalk with CRC cells that may contribute to tumorigenesis and progression. At the community level, specific microbial niches may be associated with directional regulation of this pathway via their ability to alter β‐catenin nuclear translocation and its subsequent targets (for example, AXIN2 and cellular MYC proto‐oncogene [c‐MYC]), highlighting a tight coupling between tumor‐driven cell signaling states and microbial composition [74, 75].

Beyond classical oncogenic cascades, microbe–tumor‐cell crosstalk can also reprogram disease trajectories through coordinated control of metabolic cues and intracellular stress pathways. In CRC, reshaping tumor‐associated gut microbial communities while concomitantly dampening MAPK‐driven inflammatory responses yields synergistic suppression of tumor progression, highlighting functional coupling between microbiome architecture and tumor signaling networks. In hematological malignancies, microbiota‐linked bile acid signaling can, in specific contexts, restrain tumor progression via a reactive oxygen species (ROS)–p38 MAPK–DGAT1 axis that remodels lipid homeostasis, mitochondrial function, and tumor‐associated immune phenotypes—emphasizing pronounced context dependence across tumor types and metabolic states [76, 77]. Representative studies and the associated communication modes, key pathways, and links to tumor progression are summarized in Table 2.

**TABLE 2:** Microbe–tumor cell communication in the tumor microenvironment and its impact on tumor progression.

Microbial category	Representative microorganisms	Communication molecules/signaling modalities	Target cells (tumor cells or key components of the tumor microenvironment)	Signaling pathways/mechanisms	Association with tumor progression	References
Bacteria	*Akkermansia muciniphila* (Akk)	Cyclic di‐adenosine monophosphate (c‐di‐AMP)	Intratumoral innate immune cells, including monocytes and macrophages	STING–Type I interferon–NK cell–dendritic cell axis	Remodeling the immune microenvironment and enhancing immunotherapy efficacy	[61]
Bacteria	*Fusobacterium nucleatum*	Metabolite succinate	Succinic acid receptor 1 (SUCNR1)	SUCNR1–HIF‐1α–enhancer of zeste homolog 2 (EZH2)–cGAS–IFN‐β signaling pathway	Induction of resistance to immunotherapy and promotion of tumor progression	[79]
Bacteria	*Lactobacillus reuteri*	Tryptophan metabolite indole‐3‐aldehyde	AhR on CD8^+^ T cells	AhR–CREB–IFN‐γ/Blimp‐1 signaling axis	Enhancement of anti‐PD‐L1 efficacy and suppression of melanoma progression	[80]
Bacteria	Clostridiales (e.g., Blautia)	Microbial metabolite TMAO	Tumor cell gasdermin E (GSDME) and PERK kinase	PERK–caspase‐3–GSDME‐mediated pyroptosis	Augmentation of CD8^+^ T‐cell immunity and improved immune checkpoint inhibitor (ICI) efficacy	[81]
Bacteria	Gut commensal microbiota (including *Bacteroides spp*.)	Microbial metabolites and intercellular interaction signals	Immune cells in the tumor microenvironment (T cells/ILC3)	ILC3–major histocompatibility complex (MHC) II–T cell‐mediated Type 1 immune regulation	Suppression of CRC invasion and enhancement of PD‐1 inhibitor efficacy	[82]
Bacteria	*Fusobacterium nucleatum*	CXCL2‐mediated paracrine communication	Oral squamous cell carcinoma (OSCC) cells	Activation of the nuclear factor kappa B (NF‐κB) pathway leading to CXCL2 upregulation	Promotion of OSCC proliferation, migration, and M2 macrophage infiltration	[83]
Bacteria	Akk	Mediated by CXCL3, MHC‐I/II, IFNG, etc.	Lung cancer cells, CD8^+^ T cells, and neutrophils	Inhibition of the CXCL3–PD‐L1 axis and activation of the MHC–pDC pathway	Reversal of CD8^+^ T‐cell exhaustion and enhancement of anti‐PD‐1 efficacy	[84]
Bacteria	*Lactobacillus johnsonii/*murinus/reuteri	Microbial metabolites (e.g., indole‐3‐acetic acid, I3A)	Hepatocellular carcinoma cells, Treg cells, and CD8^+^ T cells	Increase in the CD8^+^/Treg ratio and activation of the IFN‐γ pathway	Induction of tumor cell apoptosis and inhibition of hepatocellular carcinoma growth	[85]
Bacteria	*Fusobacterium nucleatum*	APRIL/BAFF/CCL3	Ligand–receptor signaling via TACI/BCMA/BAFFR/CCR1 receptors	Disruption of TGF‐β signaling and IgA maturation pathways	Increased bacterial burden exacerbates inflammation, leading to poor prognosis	[86]
Bacteria	*Lactobacillus plantarum* OC01	Short‐chain fatty acids (SCFAs) (e.g., butyrate) and other metabolites	CRC cell lines HCT116 and HT29	Downregulation of TGF‐β expression and reprogramming of macrophages toward a proinflammatory phenotype	Inhibition of tumor cell proliferation and migration, thereby attenuating cancer progression	[87]
Bacteria	*Fusobacterium nucleatum*	Outer membrane protein GTPase‐binding protein (Gbp) and CXCL8	GC cells and mast cells	Gbp–CypA–NF‐κB signaling pathway	Promotion of GC metastasis	[78]
Bacteria	*Fusobacterium nucleatum*	Connective tissue growth factor (CTGF) and other factors secreted by cancer‐associated fibroblasts (CAFs)	CAFs	TLR2–YAP–CTGF signaling axis	Promotion of tumor cell proliferation and migration	[88]
Fungi	*Saccharomyces cerevisiae*	β‐Glucan	B cells within the tumor microenvironment (indirect effect)	Dectin‐1‐dependent B‐cell activation	Enhancement of antitumor immunity and delay of tumor progression	[89]
Fungi	*Candida albicans* (specific strains/morphotypes)	Induction of macrophage metabolic reprogramming and IL‐7 secretion	Acts on ILC3 cells, indirectly affecting epithelial cells	HIF‐1α‐dependent glycolysis → IL‐7 → AhR/STAT3 → IL‐22 signaling cascade	IL‐22‐mediated STAT3 activation promotes colon tumor initiation and progression	[90]
Fungi	Hyphal form of *Candida albicans*	Cell wall β‐glucan of Candida albicans	Tumor‐associated macrophages (TAMs)	Induction of ferroptosis (ACSL4↑/glutathione peroxidase 4 [GPX4]↓) and M1 polarization in TAMs	Inhibition of lung cancer cell growth and migration, exerting antitumor effects	[91]
Fungi	*Candida albicans*	Candidalysin	Macrophages (modulating the tumor microenvironment)	Induction of macrophage pyroptosis, leading to immunosuppression	Promotion of tumor progression and exacerbation of immunosuppression	[92]
Virus	Epstein–Barr virus (EBV)	Virally encoded deubiquitinase *Bam*HI fragment leftward open reading frame 1 (BPLF1)	Cellular mTOR protein (mTORC1 complex)	BPLF1‐mediated deubiquitination of mTOR inhibits mTORC1 activation	Facilitation of EBV‐associated tumorigenesis and progression	[93]
Virus	EBV	Latent membrane protein 1 (LMP1), LMP2A, EBNA1 proteins, and EBERs	Nasopharyngeal carcinoma HONE1 cells	Suppression of JAK/STAT signaling with concomitant activation of PI3K/AKT and NF‐κB pathways	Promotion of cell proliferation and migration while inhibiting apoptosis	[94]
Virus	EBV	LMP1	Small ubiquitin‐like modifier (SUMO) gene promoter region	LMP1–CTAR1/2‐mediated activation of the NF‐κB pathway	Upregulation of SUMO expression and enhancement of protein SUMOylation	[95]
Virus	KSHV	Interaction between vBcl‐2 and NM23‐H2 proteins	Mitochondrial fission protein DRP1	Induction of mitochondrial fission suppresses MAVS signaling and interferon responses	Promotion of viral replication and assembly, facilitating tumor development	[96]
Virus	HTLV‐1	Viral proteins tax and HTLV‐I bZIP factor (HBZ) in association with host EZH2	Smad3/Smad4 transcriptional complex	Activation of the HBZ–Smad3–TGF‐β signaling axis	Promotion of regulatory T‐cell differentiation and viral persistence	[97]
Virus	HBV	Host miR‐155 regulated by viral infection	Suppressor of cytokine signaling 1 (SOCS1) protein	Suppression of SOCS1 leading to activation of the JAK/STAT pathway	Enhancement of antiviral immune responses and suppression of viral replication	[98]

Abbreviations: Akk, Akkermansia muciniphila; CAFs, cancer‐associated fibroblasts; c‐di‐AMP, cyclic di‐adenosine monophosphate; EBV, Epstein–Barr virus; EZH2, enhancer of zeste homolog 2; Gbp, GTPase‐binding protein; GPX4, glutathione peroxidase 4; GSDME, gasdermin E; HBZ, HTLV‐I bZIP factor; ICI, immune checkpoint inhibitor; MHC, major histocompatibility complex; NF‐κB, nuclear factor kappa B; OSCC, oral squamous cell carcinoma; SCFA, short‐chain fatty acid; SUCNR1, succinate receptor 1; SUMO, small ubiquitin‐like modifier; TAMs, tumor‐associated macrophages.

Importantly, a substantial proportion of these signaling changes occur in TMEs already reprogramed for immune and metabolic derangement, indicating microbial modulation of tumor‐cell signaling frequently functions as an “amplifier” rather than an “initiator.” Accordingly, many of these observations should be interpreted as model‐supported or association‐based mechanisms rather than universally established causal pathways. In the context of this model, direct molecular crosstalk represents a central regulatory layer responsible for modulating phenotypic heterogeneity and evolutionary potential, ranging from structural sensing on transmembrane signaling all the way to intracellular stress control. In this way, bacteria may directly impact on proliferation programs, drug resistance, and epithelial–mesenchymal transition (EMT) in specific contexts [78]. Subsequent studies should systematically define the key effector molecules and host receptors of interkingdom signaling, as well as their stage‐specific kinetics, to facilitate mechanism‐informed interventions that selectively disrupt individual signaling relays and thereby manipulate tumor progression.

## 4 Microbiome–Tumor Mechanisms of Initiation and Progression

The mechanisms discussed in this section are intended to summarize biologically plausible routes through which intratumoral or tumor‐associated microbiota may contribute to tumor initiation and progression. However, much of the current evidence is derived from preclinical models or associative studies, and the causal relationships between intratumoral microbiota and tumor progression remain to be fully established and may be highly context‐dependent [10, 11].

### 4.1 Microbe‐Driven Inflammatory Responses

Chronic inflammation has been commonly perceived as the biological conveyor belt connecting homeostatic microbial imbalance and oncogenesis. Taken together, an inflammatory leaning‐on across a tumor panorama not only provides enabling protumor conditions that favor tumor initiation, but may also contribute to this process through rewiring tissue homeostasis, intercellular signaling, and immune‐regulatory circuits. Tumor‐resident and tumor‐associated microbes may therefore function as more than just upstream triggers that, by adjusting the threshold, magnitude, and persistence of inflammatory stimuli, may help establish a lasting yet malleable inflammatory set point hardwired into tumor initiation and progression [27, 28, 99].

Consequently, along with other gut tumors, this partnership between microbiota and inflammation has been implicated in the predisposition to cancer. Work in genetically predisposed models has demonstrated how the gut microbiota can define the severity of colitis by regulating both the intensity and duration of inflammation, which then determines potential risk for CRC and colitis‐associated CRC (CAC); strikingly, these microbial effects correlate strongly with long‐term imprinting of innate immune cells—particularly macrophage inflammatory states as well as their anti‐inflammatory capacity [100, 101]. Crucially, microbial modulation of inflammation is not exclusively proinflammatory. Selected microbial cues have been reported to trigger and drive anti‐inflammatory programs to limit pathology such as indole‐3‐carboxaldehyde (I3A) reducing radiation‐induced gut inflammation and maintaining mucosal barrier function through activation of the AhR–IL‐10 axis [102, 103]; conversely, IL‐10 loss in a microbe‐dependent setting can critically alter metastatic progression of CRC [102, 103]. These data point to a dynamic balance between proinflammatory and anti‐inflammatory signals in the microbiota–inflammation axis, rather than an overt “protumor” pathway.

Chronic inflammation associated with microbes may promote cancer progression through the induction of immunosuppressive negative feedback loops within the TME. Chronic sensing of microbes by myeloid cells may lead to calcineurin–nuclear factor of activated T cells (NFAT) signaling driving IL‐6 production and expression of tumor‐cell coinhibitory molecules, for example, B7H3 and B7H4, suppressing CD8^+^ T‐cell‐mediated immunity. A similar rationale could be provided in *H. pylori*‐related GC, where constantly elevated IL‐1β and IL‐6 may give rise to an immunosuppressive inflammatory condition [104, 105]. Functionally, this axis is therapeutically pliable: in preclinical models, pterostilbene and astragaloside IV–PESV remodel microbial community structure and suppress inflammatory pathways (including NF‐κB), reducing inflammatory burden and potentially limiting tumor progression in colorectal and prostate cancer settings [106, 107]. Together, these studies support the microbiota–inflammation axis as a potentially important upstream environmental determinant of tumor behavior.

**FIGURE 4:**
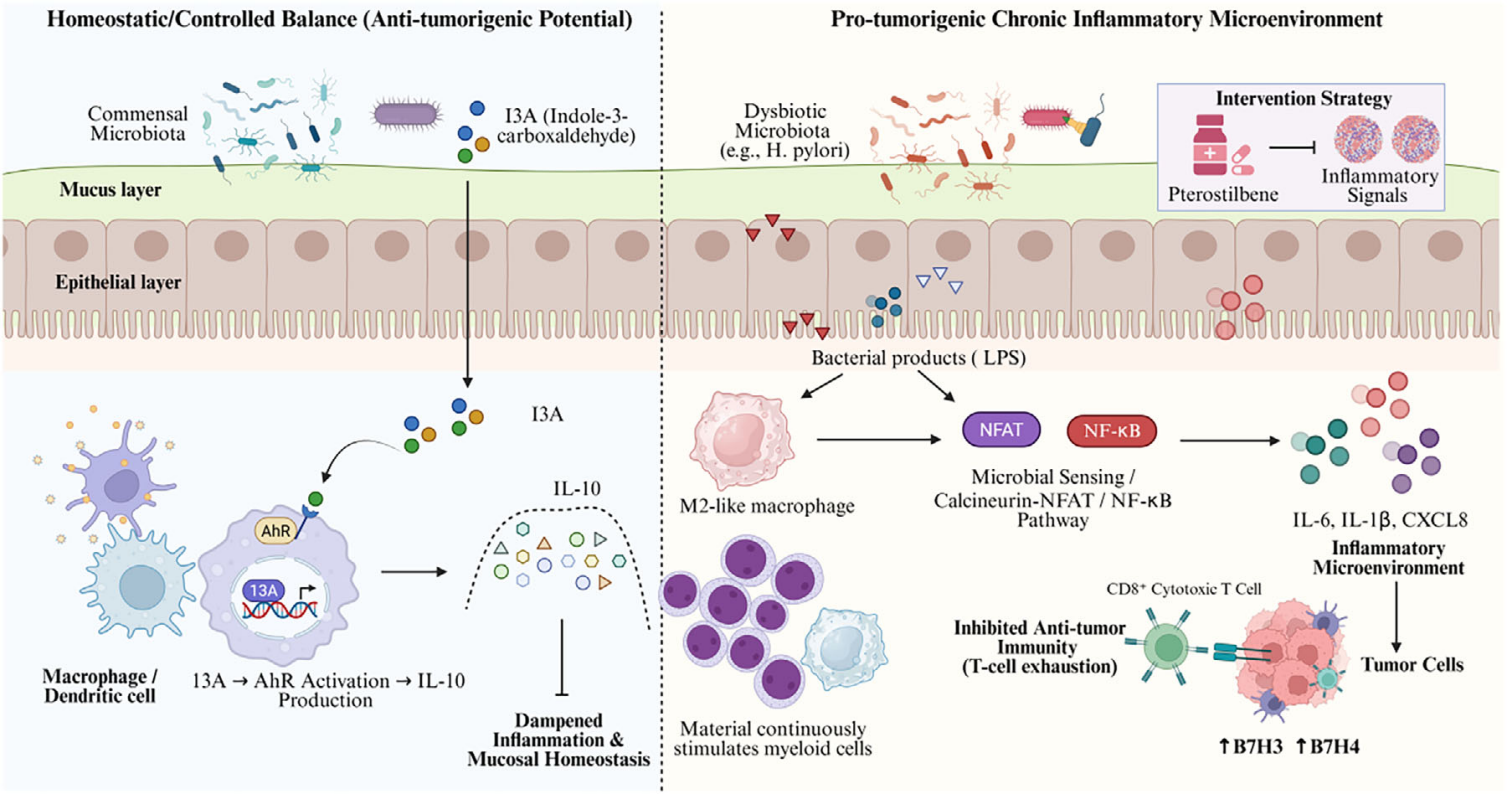
Microbiota‐associated inflammatory responses in tumor initiation and progression. Dysbiosis of the microbiota and the spatiotemporal rewiring of inflammatory signaling are crucial components of the tumor‐promoting chronic inflammatory environment. In homeostasis, resident commensals secrete ligands such as indole‐3‐aldehyde (I3A) to activate aryl hydrocarbon receptor (AhR) signaling that skews myeloid cells such as macrophages and dendritic cells toward a IL‐10‐centered anti‐inflammatory feedback program that promotes maintenance of mucosal tissue barriers and tissue homeostasis keeping in turn a functional space for an antitumoral immunity. By contrast, pathogenic or imbalanced microbiota (for example, *Helicobacter pylori*) and their products such as lipopolysaccharide (LPS) are persistently sensed by myeloid cells, activating calcineurin–nuclear factor of activated T cells (NFAT) and nuclear factor kappa B (NF‐κB) pathways and sustaining the chronic release of IL‐6, IL‐1β, and C–X–C motif chemokine ligand 8 (CXCL8). This establishes a suppressive niche in which an amplified inflammatory cascade coexists with immune tolerance, promotes tumor‐cell upregulation of coinhibitory molecules (including B7‐H3 and B7‐H4), dampens CD8^+^ T cell effector function and accelerates T cell exhaustion. Collectively, the network highlights the microbiota–inflammation axis as an upstream ecological control layer that, in a context‐dependent manner, intensifies DNA damage and immunosuppressive pressure to hasten tumor initiation, therapy resistance, and malignant progression, while remaining constrained by host genetics and the local homeostatic threshold of the microenvironment (by BioRender). AhR, aryl hydrocarbon receptor; CXCL8, C‐X‐C motif chemokine ligand 8; I3A, indole‐3‐aldehyde; LPS, lipopolysaccharide; NFAT, nuclear factor of activated T cells; NF‐κB, nuclear factor kappa B.

On the whole, through controlling initiation, maintenance, and persistence of inflammation, microbes may assist in creating tumor‐permissible or tumor‐restricting inflammatory niches. This result is consistent with context‐dependent and stage‐dependent moderation of a variety of proinflammatory and anti‐inflammatory microbe‐related phenomena, rather than one pathway dominating. Integrating these mechanisms may help refine the discovery of pivotal drivers of tumorigenesis and establish a robust biological framework for subsequent events such as microbe‐related genotoxicity, metabolic reprogramming, and signaling rewiring. Figure 4 illustrates how sustained microbial and metabolite‐driven tuning of the TME immune homeostasis threshold governs the directional antagonism between AhR–IL‐10 anti‐inflammatory feedback and NFAT/NF‐κB‐reinforced chronic inflammatory circuits, ultimately calibrating CD8^+^ T cell effector function versus exhaustion. Nevertheless, the relative contribution of microbial‐driven inflammation to tumor progression is likely to vary across tumor types and experimental contexts and requires further validation in human studies. Collectively, these mechanisms suggest that microbial influences on tumor initiation and progression operate through interconnected inflammatory, genomic, and signaling pathways in a context‐dependent manner [108].

### 4.2 Microbiome‐Driven Genomic Instability

Genomic instability is well recognized as an integral molecular hallmark of tumor onset and progression, manifested by the runaway accumulation of DNA damage, chromosomal aberration, elevated mutational burden, and disruption of genetic and epigenetic regulatory networks [28, 109]. As understanding of the intratumor microbiome has evolved, increasing evidence suggests that certain microbes and their products not only influence cancer indirectly through inflammation and immune modulation, but also have been reported to exert direct effects on host DNA damage signaling and repair pathways thereby potentially contributing to genomic instability and promoting tumor initiation and progression.

In the TME, microbe‐associated chronic inflammation frequently occurs with sustained increments in ROS and reactive nitrogen species (RNS), leading to greater burdens of oxidative DNA damage. Mismatch repair (MMR) and nucleotide excision repair (NER) are critical for genome stability but in the presence of persistent inflammatory signals, combined with impaired activity of these pathways, oxidized bases and DNA adducts are not effectively repaired, leading to continuous accumulation of mutations and subsequently elevated risk for genomic instability [101, 110]. This paradigm highlights the role of a microbe–inflammation–DNA repair axis on the generation of mutations in tumors.

In addition to inflammation‐mediated suppression of repair, the presence of some tumor‐promoting pathogens may promote genomic instability by directly causing DNA damage. For instance, *Escherichia coli* strains carrying the pks genomic island produce colibactin and induce DNA damage—especially in the form of DNA double‐strand breaks (DSBs)—upon intimate cocultivation with colorectal epithelial cells. As DSBs are initiating lesions for chromosomal rearrangements, amplification, and copy number variation increases, this mechanism suggests a potential molecular pathway to such genomic alterations [111, 112]. In GC, *Helicobacter pylori* infection provides the best‐known example of microbe‐induced genomic destabilization: it can impair repair of oxidative injuries by inhibiting expression of DNA repair proteins such as Nei‐like DNA glycosylase 2 (NEIL2), but also cause single‐strand and double‐strand breaks related to loss of checkpoint control. In the setting of environmental carcinogens, *H. pylori* additionally inhibits expression of fundamental nonhomologous end joining (NHEJ) components, prolonging DNA damage accumulation and enhancing genomic instability during gastric epithelial malignant transformation [113, 114, 115].

**FIGURE 5:**
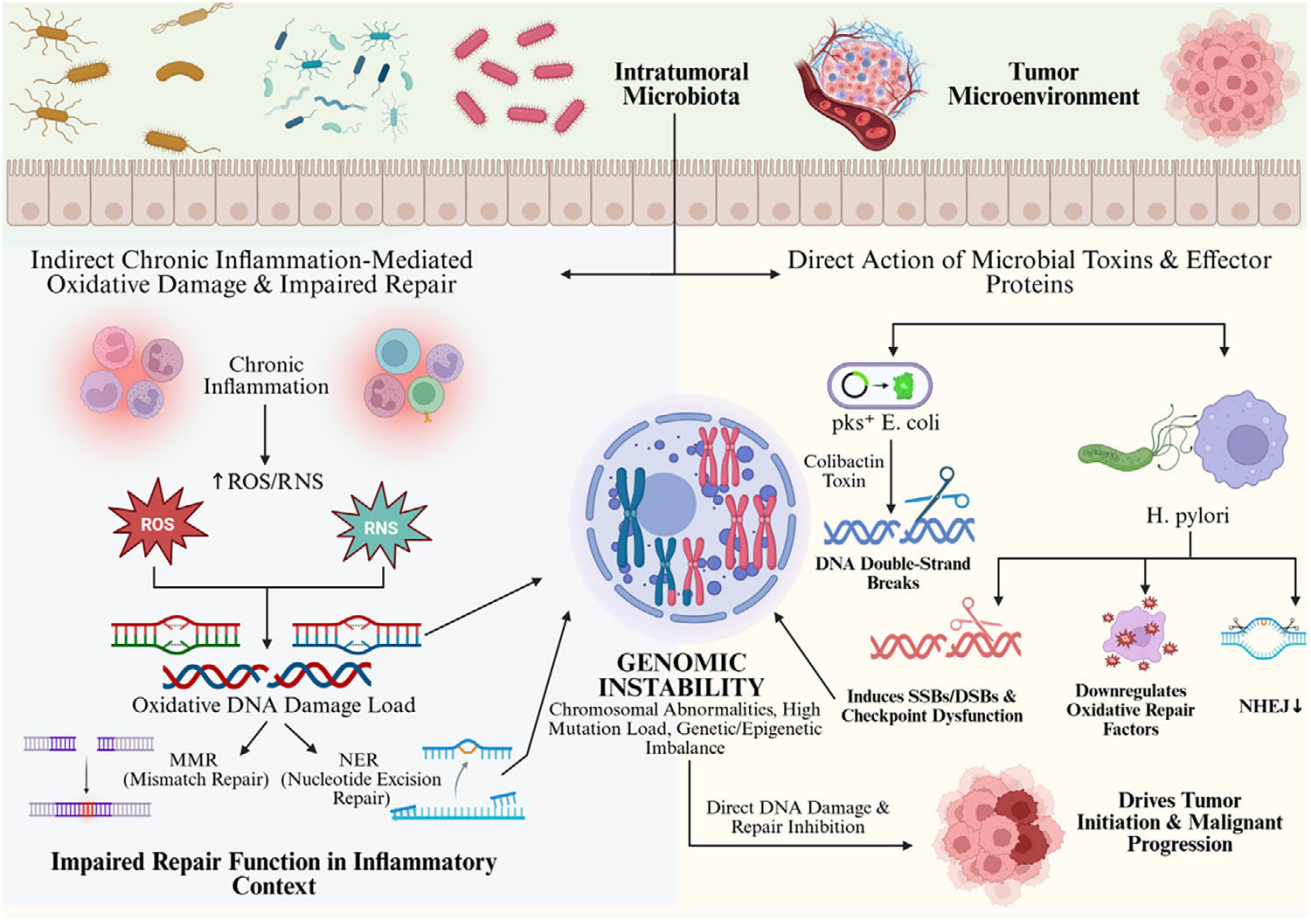
Mechanisms by which intratumoral microbes drive genomic instability in cancer. Local control of host genome homeostasis by the intratumoral microbiota can be weakened by inflammation‐induced oxidative damage and direct action of microbial toxins and effector proteins, thus enhancing and accelerating selective pressures that drive tumor evolution. This occurs indirectly through maintenance of the production of inflammatory mediators by chronically activated myeloid cells, which drive reactive oxygen species (ROS)/reactive nitrogen species (RNS) and increase in oxidative DNA damage over time that is enhanced with inhibition of mismatch repair (MMR) and NER leading to a lowered threshold for repair capacity whilst inflammation is also ongoing. Directly, colibactin produced by pks^+^ *Escherichia coli* induces DNA double‐strand breaks (DSBs), whereas *Helicobacter pylori* suppresses oxidative repair factors [for example, Nei‐like DNA glycosylase 2 (NEIL2)] and compromises key nodes of nonhomologous end joining (NHEJ), resulting in concurrent single‐strand and double‐strand breaks, checkpoint dysfunction, and impaired damage resolution. The cumulative damage increases mutational burden, chromosomal rearrangements, and copy number variation, promoting tumor initiation, immune‐checkpoint upregulation, genetic and epigenetic deregulation, and malignant progression; however, the magnitude of this contribution remains constrained by host context and the state of the tumor microenvironment (TME) (by BioRender). DSBs, DNA double‐strand breaks; MMR, mismatch repair; NEIL2, Nei‐like DNA glycosylase 2; NHEJ, nonhomologous end joining; RNS, reactive nitrogen species; ROS, reactive oxygen species; TME, tumor microenvironment.

Microbiome‐related DNA damage and repair imbalance are mainly developed against composite background of systemic inflammation, metabolism disruption, and immune remodeling, which further complicates attribution to causative effects during tumorigenesis. While robust genomic instability can be induced in well‐defined infection models or at high‐dose exposure, the level appears to depend on host genotype and microenvironmental condition. Hence, the microbial genotoxicity can be more sensibly regarded as an enhancer and expediter of pre‐existing evolutionary forces rather than a ruling cause for mutational lineages or tumor induction—hinting at the context‐mediated implication in an evolutionary facilitator stratum postulated. Accordingly, Figure 5 shows intratumor microbes as an upstream amplification tier in the TME, where ROS/RNS‐driven oxidative stress and colibactin‐induced DSB pressure are integrated with inhibition of NER elements and disruption of NHEJ nodes to promote premature genomic instability.

### 4.3 Microbe‐Induced Rewiring of Signaling Pathways

In tumor onset and progression, microbial contributions may extend beyond genomic instability and metabolic reprogramming to include the continuous remodeling of host signaling networks. A durable and biologically relevant feature of the TME, intratumoral microbes (and their structural motifs, metabolites, and secreted factors) are perceived by host cells to be transduced into signaling inputs. The outcome is co‐ordinated reprogramming of pathways underpinning inflammation, proliferation, invasion–migration, and immune response to influence tumor cell state in collaboration with evolutionary trajectories.

In microbe‐mediated immune signaling, tumor‐associated network alteration is centered on routes of inflammation. Under dysbiosis, gut microbiota have been reported to promote tumorigenesis in specific models by inducing intestinal permeability, LPS translocation in the TME, and recurrent activation of NF‐κB–IL‐6–STAT3 signaling in an orthotopic model of prostate tumorigenesis and progression [72]. This ALPK1–TIFA sensing module may be harnessed by microbial factors derived from bacteria, namely, *Fusobacterium nucleatum* in CRC, to promote activation of NF‐κB signaling in tumor and nontumor epithelia (e.g. intestinal) cells where it upregulates expression of inflammatory genes such as ICAM1, perpetuating a protumorigenic state of inflammation and modulating the local environment [116, 117]. Taken together, these findings support a model in which chronic, low‐grade inflammation progressively evolves from a defensive program to being hijacked as a signaling network that may promote tumor growth.

In addition to inflammation, microbe‐associated signals can also directly act on intrinsic tumor circuits that govern growth, survival and invasion. Several studies suggest that modifying tumor‐associated gut microbial communities may concomitantly modulate classical oncogenic pathways, including PI3K–AKT, MAPK, and Wnt/β‐catenin with functional effects on cancer progression. For instance, natural product SC‐1 inhibits the progression of CRC possibly through the reshaping of gut microbiota and inhibition of MAPK‐mediated inflammatory activation [76]. Through the promotion of butyrate producing bacteria, ECH suppresses PI3K–AKT and EMT, thereby exhibiting strong antimetastatic activity upon oral administration [118]. Additionally, POE exerts tumor‐suppressive activity by blocking the Wnt/β‐catenin–c‐Myc/cyclin D1 cascade, and nodakenin is a dual‐targeted agent against STAT3 and Wnt/β‐catenin signaling pathways that promotes microbial restoration in multiple models of CRC [119, 120].

While the microbe‐mediated signaling rewiring presents a useful model for interpreting tumor evolution, the evidence is internally in tension. In particular, many reported signaling effects derive from intervention studies in model systems, and their generalizability to human tumors remains to be clarified. Contemporary studies often report simultaneous changes—inflammatory activation, enhancement of growth pathways, and immune reprogramming—but seldom address the directionality or hierarchy of these processes. Furthermore, signaling remodeling commonly accompanies genomic instability and metabolic reprogramming, indicating systems‐level coupled adaptation rather than an unidirectional microbial push. Indeed, among tumor types and host contexts, microbe‐associated signals can support progression or induce immune homeostasis; the context dependence is very marked. This functional ambivalence refutes the dogmatic classification of microbes into oncogenic and tumor‐suppressive categories and places them as a dynamic “signaling modulation layer” rather than a deterministic driver of initiation.

## 5 Microbes as Tumor Biomarkers

This section outlines the emerging role of microbiota as tumor biomarkers, including their applications in diagnosis, prognostic stratification, and methodological approaches for microbial detection.

### 5.1 Microbiome‐Based Diagnostics

Improvements in high‐throughput sequencing and spatially resolved microbial profiling are increasingly uncovering intratumoral microorganisms with tumor‐associated microbial signatures as candidate biomarkers with potential clinical importance. In contrast to traditional diagnostic approaches that rely on the presence of a single molecular alteration or driver mutations, microbiome readouts integrate coordinated changes in community structure, composition, and functional potential and thus represent an ancillary but dynamic tool for monitoring the biological state within TME. In theory, such multidimensional data could enrich established models of tumor initiation, progression, and molecular stratification, providing novel factors to assess for early detection and risk prediction.

Evidence is increasing that a variety of solid tumors and hematological malignancies are enriched for semi‐stable microbial communities. These distinctions also transcend global diversity and abundance patterns and seem to be modulated by tumor type, stage, and primary tissue. Benign disease and ovarian cancer in gynecological cancers demonstrate marked differences in microbiome composition and fewer levels of diversity at multiple sites, highlighting that a number of putative pathogenic taxa were enriched for low‐grade early‐stage ovarian cancer, thus reinforcing the potential applicability of microbial features for early detection and population screening [121]. Likewise, systematic interrogation of TME microbial signatures has enabled the nomination of candidate diagnostic markers for HPVI ECA, expanding the prospective role of microbiome biomarkers in molecular subtyping and differential diagnosis within specific tumor entities [122].

In gastrointestinal cancers, microbiome‐derived biomarkers from stool and other biofluids have shown particularly strong diagnostic promise. Multiple studies link gut dysbiosis to CRC development and progression, providing a rationale for microbiome‐informed diagnostics [123]. During CRC evolution, stage‐associated fecal and salivary microbial profiles appear to shift dynamically; these patterns can discriminate tumor versus nontumor states and may serve as noninvasive biomarkers for identifying early, resectable CRC and for tracking disease trajectories [124]. At the level of specific applications, combined detection of *Shigella flexneri C.11* and its metabolites distinguished CRC cases from healthy controls with an AUC of 0.887 in the reported cohort, underscoring the potential of integrated microbial–metabolite signatures for noninvasive detection [125].

Additionally, cross‐disease diagnostic value is arising outside of individual tumor types. For example, *Bacteroides* is one candidate biomarker among HIV‐positive patients with a history of multiple cancers, indicating that the microbiome features may continue to bear disease‐informative signature despite intricate immunological environment [126]. Moreover, multispecies bacterial panels have demonstrated relatively consistent performance when applied across tumors (AUC 0.840 in CRC, 0.747 in non‐small cell lung cancer [NSCLC]), suggesting the feasibility of interpreting pan‐cancer microbiome‐enabled detection—albeit with the caveat that generalizability has yet to be confirmed within larger and more heterogeneous populations [127].

**FIGURE 6:**
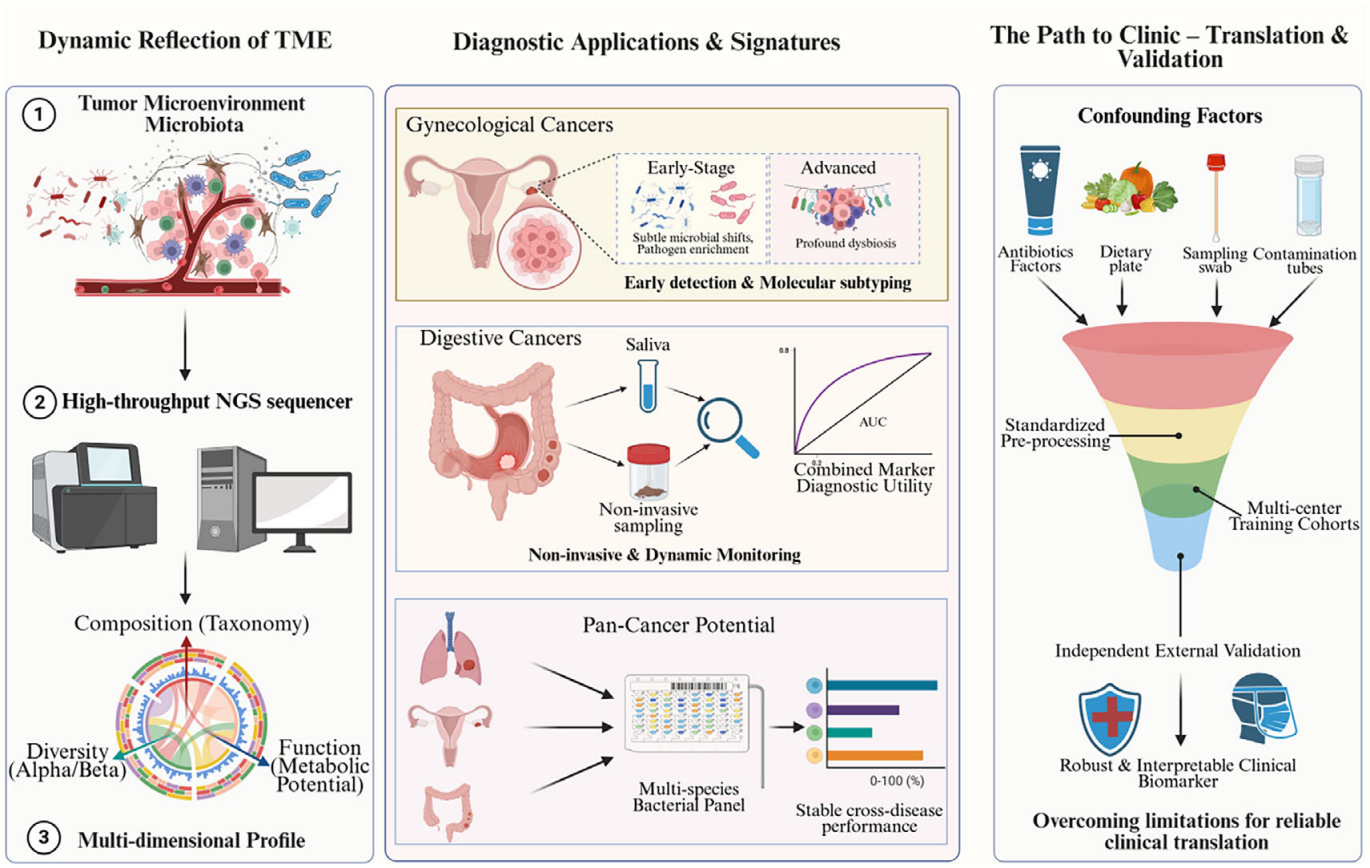
Microbiome‐based signatures and mechanisms for tumor diagnosis. Cancer diagnosis using microbiota‐based approaches has been moving from simple taxonomic profiling to the integrative exploration of microbial functions and mechanisms. A three‐staged, stepwise figure is presented. At the initial discovery stage, diagnosis is based on culture or 16S rRNA sequencing but these methods are limited due to low microbial biomass and noise‐driven biases; consequently, there is poor functional information derived. The next high‐throughput, multiomics phase incorporates metagenomic fragments, spatial‐transcriptomics, and metabolomic profiling to serve as a highly‐resolved functional inference in addition to spatial localization of tumor associated microbial signatures. In the mechanistic frontier stage, single‐cell sequencing, fluorescence in situ hybridization (FISH), and organoid coculture models are additionally integrated to define causal strain–host interaction pathways and attain stringent functional validation. In gynecologic malignancies, diagnostic applications utilize microbial signaling molecules, such as indole‐3‐aldehyde (I3A) and IL‐33, for early diagnosis and molecular subtyping. In cancers of the digestive system, saliva‐ and other noninvasive specimen‐based dynamic monitoring for metabolic and microbial biomarkers in relation to longitudinal risk estimation. From a pan‐cancer standpoint, multivariate bacterial panels show consistent, cross‐tumor generalizability in cancer types including colorectal cancer (CRC) and non‐small cell lung cancer (NSCLC), supporting an empirical basis for interpretable, well‐performing, and clinically applicable microbiota‐derived biomarkers (by BioRender). CRC, colorectal cancer; FISH, fluorescence in situ hybridization; I3A, indole‐3‐aldehyde; NSCLC, non‐small cell lung cancer.

In broader perspective, the intratumoral microorganisms and tumor‐associated microbiome signatures are starting to evolve from descriptive observations into potential translational diagnostics due to their tissue specificity, stage association, and technical determinability. However, evidence to date is largely correlative and confounded by antibiotic or acid‐suppressant exposure, diet, sampling site, tumor stage and treatment history, reagent or environmental contamination in low‐biomass settings, and batch effects. Clinical translation should therefore emphasize multicenter training–validation–external validation workflows; harmonized preanalytical processing, contamination control, and batch correction; and systematic assessment of thresholding, cross‐platform agreement, cost, and turnaround time to enhance clinical utility and interpretability. As highlighted in Figure 6, the field is shifting away from taxonomic inventories to functional readouts with causal validation—aimed at generating clinically interpretable, actionable evidence for early cancer detection, molecular characterization, and spatial localization.

### 5.2 Microbial Markers for Prognostic Stratification

Whereas tumor biomarkers have evolved from detection to clinical implication, microbiome investigations have also advanced beyond diagnostic taxonomy to predict disease course and therapeutic responses. In addition to diagnostic potential under study, intratumoral/tumor‐associated microbiota are now attractive candidates for risk classification. Emerging evidence suggests that microbial composition, diversity, and functional status may reflect a range of biological characteristics in the TME and may also accompany tumor kinetics, risk of recurrence, and survival, making the microbiome an informative layer for prognosis and monitoring disease progression. It is worth noting that prognostic biomarkers are essentially risk factors associated with the natural history of disease, whereas predictive biomarkers describe the probability for benefit from a specific therapy, yet in the microbiome field these two purposes/aims often get conflated and are therefore discussed together rather than as distinct entities. Microbiome signals from tumor tissue and local sources are biologically defined by the cancer ecological niche, whilst signatures in biofluids or gut measurements more likely reflect systemic influences; these data streams vary in terms of spatial attribution and interpretive scale and should thus be analyzed separately.

Over various solid and hematological cancers, certain taxa and community profiles have been consistently related to outcomes. In CRC, advanced TNM stage and intratumoral *Fusobacterium nucleatum* positivity are associated with worse survival. An enrichment of Enterobacteriaceae in the microbiome is also associated with early relapse and poorer progression‐free survival in ENKTL, indicating that the microbiome may serve as a surrogate read‐out for tumor aggressiveness and/or a risk state for microbiome–immune interactions [128, 129]. Prognostic models based on community‐level signatures are more robust and discriminative than single‐taxon markers. Metagenomic sequencing permits comprehensive profiling at the species, gene, and pathway levels and offers a technical infrastructure conducive to implementing microbial communities and their functional profiles as outcome biomarkers in CRC, especially in the context of large multicenter studies [130]. Clinically applicable diversity metrics contain prognostic and predictive information as well. For example, the PD (α‐diversity) of Faith's has been described as a predictive and prognostic biomarker in metastatic TNBC patients treated with chemotherapy plus immunotherapy; higher diversity was associated with better outcomes [131].

Treatment‐related prognostic evaluation is also based on microbiome characteristics. Both gut and intratumoral microbial states have been linked with the efficacy of immunotherapy and long‐term survival after immune checkpoint inhibitors (ICIs). Physiologic colonic ^18^F‐FDG uptake on PET/CT has been associated with clinical outcome in advanced NSCLC and higher diversity of the gut microbiome, so integrating imaging‐based markers with microbiome readouts may improve predictive value [132]. Furthermore, lower respiratory tract multiomics profiling has the potential to predict durable responses to checkpoint blockade in advanced stage NSCLC while gut microbiome composition and functional indices have been identified as independent predictors of immunotherapy response [133, 134].

Because the microbiome is both plastic and dynamic—its composition and function can change during tumor evolution and with therapeutic perturbation—longitudinal profiling could potentially allow real‐time monitoring of progression, of relapse risk, and of responsiveness to treatment. By incorporating microbiome signatures into classical clinicopathological features, imaging metrics, and multiomics strata, more accurate prediction models may help inform new tools and frameworks for precision oncology.

### 5.3 Detection Methods for Microbial Biomarkers

For the clinical implementation of microbiome‐based tumor biomarkers the reliability, accuracy, and reproducibility of these assays are crucial. Secondary to the development of high‐throughput sequencing, as well as spatially resolved profiling, various methods have been developed for the interrogation of intratumoral and tumor‐associated microbes with their own unique strengths and weaknesses. From a methodological point of view, intratumoral microbiome identification greatly resembles that of the gut microbiome workflow and generally involves three components: nucleic‐acid‐based community profiling, tissue‐ and spatial‐localization assays, as well as multiomics integrating computational analytics [135]. Because different biospecimens capture different biological strata, detection strategies should be stratified by sample type: tumor or adjacent tissue is best suited to confirming colonization and spatial interactions, whereas liquid biopsies are more appropriate for noninvasive screening and longitudinal monitoring.

The most commonly used platforms for compositional and diversity profiling are 16S rRNA amplicon sequencing and shotgun metagenomics. Despite its disadvantages, 16S rRNA sequencing is relatively cost effective and operationally mature for large cohort studies [136]; however, it suffers from limited resolution at the taxonomic level, limited functional inference, and incomplete coverage of taxa not belonging to bacteria. In contrast, metagenomic sequencing allows system‐wide interrogation at the levels of species, gene and pathway, and represents a crucial technical underpinning for progress from descriptive associations to mechanistic understanding and clinical translation [135, 136]. Moreover, combined culturomics–metagenomics pipelines have been applied to systematically catalogue microbes in tumor tissue, offering a practical framework for downstream biomarker discovery [135].

Detection at tissue level is being focused more and more. In situ hybridization, immunohistochemistry and laser‐capture microdissection with sequencing can also be used to map microbial localization while retaining tissue architecture to distinguish true intratumoral residents from exogenous contaminants and resolve spatial relationships with tumor cells as well as immune cells [136]. Concurrently, liquid‐based tests would offer an important adjunct for screening and longitudinal monitoring; in particular, optimizing the extraction of microbial DNA from urine could make a substantive difference to the robustness of analysis within studies relating to bladder cancer‐associated urinary microbiome [137].

Third‐generation long‐read sequencing (for example, MinION) confers additional power for the resolution of complex communities and has started to widen the analytical toolkit for studies of intratumoral microbiomes [138]. To establish a structured summary of tumor‐associated microbes as potential markers in cancer, including the status of studies, specimen types, and analysis platforms used, an overview was compiled from relevant publications; examples are presented in Table 3. The current microbial biomarker pipelines are transitioning from single sequence‐heavy methods to multitechnology assays, tiered validation, and integrated multidimensional data models. At the moment, most clinically applicable tests are based on quantitative polymerase chain reaction (qPCR) and targeted sequencing, whereas shotgun metagenomics and spatially resolved strategies are primarily used for the discovery of biomarkers and mechanistic support. A major methodological challenge of clinical translation is to simultaneously establish and improve sensitivity and specificity while promoting robust standardization in the handling of specimens, sequencing pipelines, computational analysis, and result interpretation, to enable cross‐study comparison as well as reproducibility. Together, these findings suggest that intratumoral microbiota hold promise as biomarkers for tumor stratification and therapeutic prediction, although their clinical utility remains to be further validated [16].

**TABLE 3:** Overview of studies on tumor‐associated microorganisms as biomarkers.

Biomarker type	Microbial characteristics	Sample source	Detection method	Associated tumor type	Clinical relevance	Reference
Multikingdom microbial species and functional gene biomarkers	Combined multikingdom features of bacteria, fungi, and archaea	Fecal samples from patients and healthy controls	Shotgun metagenomic sequencing with quantitative PCR validation	Colorectal cancer (CRC)	Applied for noninvasive early diagnosis of CRC	[139]
Microbial species, metabolites, and functional gene biomarkers	Multikingdom microbial combinations and specific pathogens	Fecal samples from patients and healthy controls	Metagenomic sequencing with predictive metabolomic analysis	CRC	Used for noninvasive diagnosis and early detection	[140]
Functional fecal microbiota transplantation	Whole gut microbiota capable of modulating antitumor immunity	Gut microbiota from anti‐PD‐1–refractory melanoma patients	Fecal microbiota transplantation combined with immune and gene expression analyses	Metastatic melanoma	Overcomes immunotherapy resistance and reinduces clinical responses	[141]
Tumor location‐specific microbial species biomarkers	Site‐specific microbial assemblages and abundance gradients	Fecal samples from patients and controls	Fecal metagenomic sequencing	Right‐sided, left‐sided, and rectal CRC	Used for site‐specific diagnosis and prognostic evaluation	[142]
Functional microbiota transplantation	Whole gut microbiota capable of modulating antitumor immunity	Gut microbiota derived from patients with PD‐1–refractory melanoma	Fecal microbiota transplantation (FMT) integrated with multiomics analyses	Advanced melanoma	Overcoming PD‐1 resistance and reinducing therapeutic responses	[10]
Cross‐kingdom microbial biomarkers	Specific bacterial and eukaryotic species enriched in responders	Fecal samples from cancer patients treated with immune checkpoint inhibitors (ICIs)	Fecal metagenomic sequencing combined with machine learning modeling	Melanoma, non‐small cell lung cancer, and renal cell carcinoma	Predicts patient responses to ICIs	[143]
Intratumoral microbiome	Diversity and abundance of bacteria and fungi	Tumor tissues	Optimized microbial analysis pipeline minimizing host sequence interference	Thirty‐three cancer types	Associated with tumor immune phenotypes, prognosis, and cancer types	[20]
Stage‐ and site‐specific microbial species and functional biomarkers	Enrichment of oral bacteria, unidentified species, and strain‐level genetic variations	Large‐scale, multicohort fecal samples from patients and controls	Metagenomic sequencing with advanced strain‐level bioinformatic analyses	CRC	Used for noninvasive diagnosis and monitoring of disease progression	[144]
Tumor‐associated fungal DNA	Candida species associated with proinflammatory immune pathways and metastasis	Tumor tissues from gastrointestinal cancers	ITS sequencing combined with culture‐dependent analyses	Lung cancer, gastric cancer (GC), and colon cancer	Indicative of reduced survival and increased cancer metastasis	[13]
Functional metabolites and regulatory microbial biomarkers	Enrichment of butyrate‐producing bacteria and increased microbial diversity	Fecal samples from humanized mouse models	16S rRNA sequencing and metabolomic analysis (GC–MS)	CRC	Enhances the efficacy of anti‐PD‐1 immunotherapy and overcomes resistance	[145]
Tissue‐resident microbial species biomarkers	Coabundance clusters of Fusobacterium and *Bacteroides*	Tumor, adjacent normal tissue, and blood samples from the cancer genome atlas (TCGA) patients	Multiplatform sequencing integrated with statistical modeling and validation	Colorectal and other gastrointestinal cancers	Used to predict host gene expression, prognosis, and disease mechanisms	[146]
Combined microbial species and serum biomarkers	Specific microbial species enriched or depleted in pancreatic ductal adenocarcinoma (PDAC)	Multisite samples, including feces, from PDAC patients and controls	Shotgun metagenomic sequencing combined with machine learning models	PDAC	Used for early noninvasive screening and diagnosis	[147]
Diagnostic microbial biomarkers	Altered abundance of genera such as Blautia	Fecal samples from patients	16S rRNA gene V3–V4 region sequencing	Thyroid cancer (papillary carcinoma)	Significantly associated with tumor T stage	[148]
Prognostic microbial biomarkers	High intratumoral bacterial load and specific dominant genera	Biopsy tissue samples from nasopharyngeal carcinoma patients	16S rRNA sequencing and quantitative PCR	Nasopharyngeal carcinoma	Significantly associated with poor prognosis and reduced survival	[32]
Gut microbiota as risk prediction biomarkers	Rikenellaceae Increased abundance of Rikenellaceae RC9 and decreased Lactobacillaceae and Ruminococcus 2	Fecal and blood samples	Mendelian randomization and genome‐wide association analyses	CRC	Predicts CRC risk in patients with inflammatory bowel disease	[149]
Gut microbial species biomarkers	Increased abundance of six potential biomarker species	Fecal and serum samples	Nanopore third‐generation sequencing and ELISA	CRC	Potential noninvasive diagnostic biomarkers	[150]
Adenoma‐associated gut microbiota biomarkers	Specific bacterial consortia and altered metabolic functions	Fecal samples from multicohort CRC patients	Metagenomic analysis combined with quantitative real‐time PCR	Colorectal adenoma and CRC	Used for early detection of adenomas and discrimination from malignant transformation	[151]
Gastrointestinal tumor‐associated fecal bacterial biomarkers	Reduction of multiple beneficial bacteria and enrichment of opportunistic pathogens	Fecal samples from patients with esophageal, gastric, and CRC	16S rRNA sequencing combined with functional prediction analyses	Esophageal cancer, GC, and CRC	Used for noninvasive differential diagnosis of gastrointestinal cancers	[152]
Intratumoral oral microbiome composition characteristics	Stage‐specific alterations in the abundance of distinct bacterial genera	Biopsy samples of oral squamous cell carcinoma (OSCC) tissues	16S rRNA amplicon sequencing combined with immunological analyses	OSCC and its premalignant lesions	Associated with tumor stage and immune infiltration status	[153]
Functional biomarkers of gut microbiota and their metabolites	Acarbose‐modulated microbiota and tryptophan metabolism	Gut and tumor tissues from tumor‐bearing mouse models	Microbiota intervention experiments combined with immune functional analyses	Experimental tumor models sensitive to immune checkpoint therapy	Enhances anti‐PD‐1 efficacy and increases T‐cell infiltration	[154]
Tumor‐associated mucosal microbial biomarkers	Specific bacterial genera significantly correlated with differentially expressed genes	Intestinal mucosal tissue samples from CRC patients	Transcriptomic sequencing integrated with microbiome bioinformatic analyses	CRC	Significantly associated with tumor‐related gene expression regulation	[155]
Inflammation‐associated airway microbial biomarkers	Enrichment of specific bacterial genera and reduced microbial diversity	Spontaneous sputum samples	16S rRNA gene sequencing	Non‐small cell lung cancer	Significantly associated with systemic inflammatory status	[156]
Fecal microbiota composition‐related biomarkers	Significantly increased abundance of specific differential genera	Fecal samples from GC patients and healthy controls	Fecal microbiome sequencing combined with quantitative polymerase chain reaction (qPCR) validation	GC	Used for diagnostic prediction and staging correlation of GC	[157]
Combined fecal microbiota and serum biomarker signatures	Marked enrichment or depletion of genera associated with CRC progression	Fecal samples from CRC patients, polyp patients, and healthy individuals	16S rRNA sequencing combined with random forest modeling	CRC	Used for CRC staging assessment and noninvasive diagnostic prediction	[158]
Prognostic biomarkers for predicting patient survival outcomes	A microbial signature composed of six key genera, including Synergistes	Tumor tissue samples from bladder cancer patients	miRNA sequencing combined with bioinformatic analyses	Bladder cancer	Used to construct a risk scoring system for patient stratification and prognostic evaluation	[159]
Carcinoembryonic antigen (CEA)	Gut microbiota dysbiosis, particularly enrichment of *Enterococcus hirae* in patients with elevated CEA levels	Fecal samples and tumor tissues from CRC patients	16S rRNA sequencing, tumor tissue transcriptomic sequencing, and LEfSe analysis	CRC	Gut microbiota dysbiosis is associated with immune microenvironment alterations and pathway regulation during CRC progression	[160]

Abbreviations: CEA, carcinoembryonic antigen; CRC, colorectal cancer; FMT, fecal microbiota transplantation; GC, gastric cancer; ICIs, immune checkpoint inhibitors; OSCC, oral squamous cell carcinoma; PDAC, pancreatic ductal adenocarcinoma; qPCR, quantitative polymerase chain reaction; TCGA, The Cancer Genome Atlas.

## 6 Microbiome‐Modulated Strategies for Cancer Therapy

As tumors are increasingly understood as complex ecosystems comprising malignant cells, immune populations, stromal elements, and resident microbes, the microbiota is being reframed from a passive correlate of tumorigenesis to a tractable therapeutic variable. Building on the preceding discussion of microbe–immune–metabolic crosstalk, this section focuses on the functional deployment of microbes in cancer therapy, emphasizing the harnessing of these organisms to tune antitumor immunity, enable targeted drug delivery, and remodel treatment‐conditioned microenvironments to augment existing modalities. Current microbiota‐informed strategies primarily act by potentiating responses to immunotherapy, improving the precision of drug delivery and synergistically shaping outcomes of conventional treatments—underscoring a shift from “background noise” to an intervenable regulatory layer within the therapeutic landscape. Nevertheless, most microbiota‐informed therapeutic strategies remain at the preclinical or early translational stage, and their efficacy, safety, and reproducibility require further validation [10, 11].

### 6.1 Microbe‐Based Immunotherapies

Immunotherapies, particularly ICIs, have had a significant impact in the management of several cancers. However, there is a marked heterogeneity in clinical benefit with patients’ response to treatment, highlighting that the antitumor immunity is not dictated by tumor‐intrinsic genetic characteristics only. There is a growing body of evidence to suggest that tumor‐associated microbes and their metabolites can modulate systemic immune tone and antigen presentation by the immune cells, as well as the fitness of these immune cells, while intratumoral microorganisms might function within localized niches to maintain inflammatory circuits and to re‐engineer the immune microenvironment. In combination, these effects can influence both the probability and durability of response to immunotherapy. In this context, microbiome‐informed or microbiome‐targeted immunotherapies have been proposed as potentially promising strategies to address this problem.

At the intersection of effector immunity and immunometabolism, some emerging evidence indicates that certain tumor‐associated microbe and microbe‐derived metabolites may promote CD8^+^/Tc1‐associated programs to stimulate cytotoxic responses and potentiate checkpoint blockade. In melanoma and other models, microbial signals—through tryptophan catabolism and short‐chain metabolite‐mediated signaling—appear to reprogram effector T cell states and increase antitumor immunity in preclinical models, thereby improving ICI efficacy [80, 161]. In keeping with these findings, in models of PDAC, the gut‐derived metabolite trimethylamine N‐oxide (TMAO), when coadministered with anti‐PD‐1 and/or anti‐Tim3 therapy, resulted in a reduction in tumor burden and improved survival over single‐agent treatment [162]. Moreover, the immunoregulatory activities of these metabolites are highly context dependent and tumor type specific, and the systemic metabolic implications and safety profile of these mediators remain to be studied.

Microbiome‐defined immunotherapies may improve responses in selected tumor settings across the spectrum of such responses through modulation of effector‐cell function, immunometabolic set‐points, and systemic immune homeostasis. Yet, results are collectively influenced by the structure of microbial communities, host immune configuration and therapeutic context. This will require a more nuanced understanding of microbe–immune–tumor interactions to determine where such strategies are most likely to apply, and by what means efficacy is imparted.

### 6.2 Microbiome‐Associated Drug‐Delivery Systems

Clinical anticancer agents usually encounter low tumor selectivity, nonspecific biodistribution, and dose‐limited systemic toxicities upon in vivo administration, which limits the therapeutic window and compromises the clinical safety. With a better understanding of the spatial structure and chemical make‐up of the TME, microbes and microbe‐associated structures are now recognized as ideal units for creating DDS, due to their ability to target tumor‐affiliated colonization, chemotactic behavior, as well as actions that can be engineered. Hence, the variety of microbe‐mediated delivery strategies has expanded to consist of live or attenuated microbial vectors, microbe‐derived constructs, and hybrid systems in association with nanomaterials [163].

Among microbe‐based platforms, bacterial outer membrane vesicles (OMVs) have been of interest due to their biocompatibility, natural cargo capacity, and high tunability. OMVs are capable of shuttling proteins, nucleic acids, and small‐molecule metabolites while largely maintaining parent membrane architecture and interfacial cues, which promote effective crosstalks in TME. These attributes have made OMVs attractive platforms for delivery of tumor therapeutics and immunomodulatory cargoes [164, 165, 166]. Significantly, OMVs are also a prominent rather than unique example of microbe‐driven delivery platforms.

Molecular and membrane‐structural level engineering can be applied to enhance OMV cancer cell targeting as well as functional plasticity, leading to multimodal therapies. For example, surface display of PD‐1 or development of hybrid membranes that integrate tumor‐cell membranes can increase the recognition of PD‐L1–high tumors for targeted delivery to favorably deliver nucleic‐acid cargos and immunoregulatory agents while minimizing off‐target exposure. In addition to targeting, endowing peroxidase‐like activity, codelivering immune adjuvants, or using inflammation‐homing immune cells (e.g., neutrophils) as cellular shuttles to couple local ferroptosis induction with immune activation can provide additional delivery routes for postoperative recurrence control and combination immunotherapy [165, 166, 167, 168].

In addition, microbe‐induced delivery systems offer a potentially unique design rationale to overcome poor targeting and systemic toxicity in anticancer therapy, leveraging specific microbe–TME interactions. Nonetheless, it is currently unclear whether in vivo accumulation and therapeutic output still depend on tumor heterogeneity, host immune status, and dosing regimens while bearing little delivery stability or predictability. The majority of experimental data is still based on short‐term in vivo animal studies, and long‐term clearance kinetics and safety as well as repeated dosing have not been closely examined, which must be improved before clinical translation.

### 6.3 Microbial Synergy With Conventional Therapies

While surgery, cytotoxic chemotherapy, and radiotherapy continue to form the cornerstone of treatment for several cancers, their efficacy is often compromised by acquired drug resistance, cumulative toxicities, and adaptive remodeling of the TME. Data generated in recent years suggest that tumors, particularly those of gut origin, are not merely passive passengers of therapeutic perturbation but instead may actively modulate sensitivity to standard modalities by regulating metabolic programs, inflammatory signaling, and antitumor immunity. This evolving understanding expands the explanatory framework beyond tumor‐cell–intrinsic susceptibility to a systems‐level perspective that incorporates microbiome–tumor–host crosstalk, thereby positioning the microbiome as a potentially clinically relevant modifier of outcomes with standard therapies.

On the level of treatment potentiation, clinical and translational research now suggests that microbiome status may significantly influence responses to chemotherapy as well as to chemo–immunotherapy doublets. Fecal microbiota transplantation (FMT), by restructuring tumor‐related gut ecosystems, has been shown in preclinical and limited clinical settings to enhance antitumor immune activity and may improve the combined efficacy of chemotherapy plus immunotherapy. In patients with NSCLC treated by chemo–immunotherapy, treatment with a known live biotherapeutic (e.g. *Clostridium butyricum* MIYAIRI *588* [CBM588]) has been related to longer overall survival; in addition, enrichment of genera such as *Butyricicoccus* and *Fusicatenibacter* have also been linked to beneficial survival profiles [169, 170, 171]. In addition to overall gut community changes, functional pairing of specific taxa and effector immunity also buttresses mechanistic synergy: increased gut *Streptococcus* enrichment correlates with greater intratumoral infiltration of GrzB^+^ cells and CD8^+^ T cells, active responses to anti‐PD‐1 therapy can be rescued by FMT or targeted colonization. Concomitantly, microbiota‐derived SCFAs are also emerging as mediators of chemo–immunotherapy efficacy that connect with immune‐cell state and microenvironmental homeostasis as potential targets for enhancing the therapeutic responses in NSCLC and other tumors [16, 172].

**FIGURE 7:**
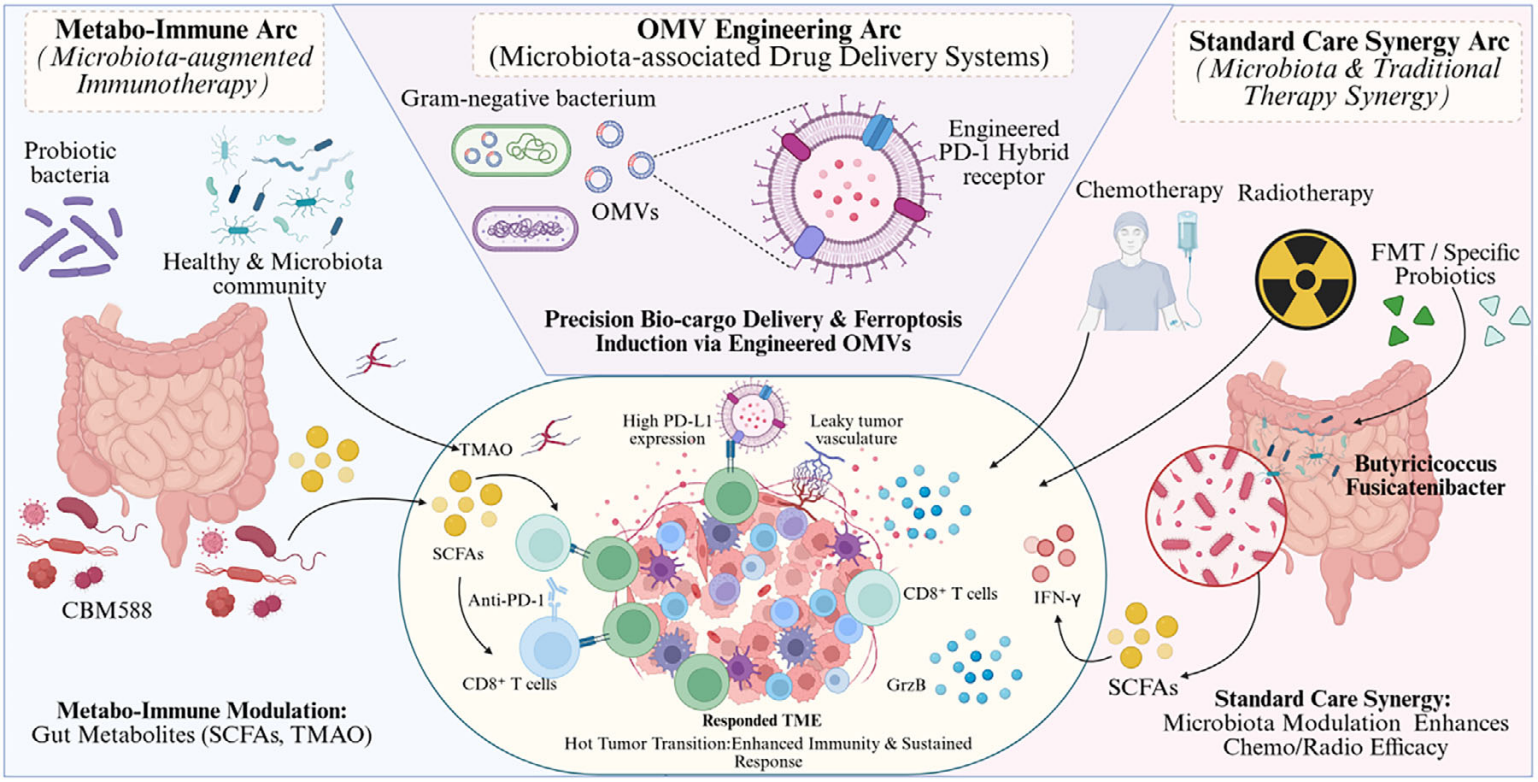
Mechanistic framework for tumor therapies that target the microbiota. Microbe‐directed delivery technology, when paired with standard‐of‐care approaches, is reshaping the mechanistic landscape of cancer treatment. The drawing suggests three strategic arcs. In the immunometabolic potentiation arc, probiotic consortia [e.g., Clostridium butyricum MIYAIRI 588 (CBM588)‐associated communities)] secrete metabolites such as short‐chain fatty acids (SCFAs) and trimethylamine N‐oxide (TMAO) to modulate PD‐1 signaling and myeloid metabolic homeostasis, thereby supporting CD8^+^ T cell function and promoting an immune‐inflamed tumor phenotype. In the engineered OMV delivery arc, gram‐negative bacterial outer membrane vesicles (OMVs) are repurposed as precision biocarriers and engineered with PD‐1‐targeting receptors for localized delivery, activating ferroptosis or cytotoxic cascades alongside immune‐checkpoint blockade. In the synergy arc with standard therapy, fecal microbiota transplantation (FMT) and defined probiotics (including butyrate‐producing clostridial taxa and Fusicatenibacter‐related groups) enhance SCFA production, reduce immunotolerance thresholds in treatment‐sensitive niches, and strengthen therapeutic impact. Overall, microbes may act as upstream modulators of the tumor microenvironment (TME), influencing immune effector activity, metabolic rewiring, and response to standard treatments in a context‐dependent manner (by BioRender). CBM588, Clostridium butyricum MIYAIRI 588; FMT, fecal microbiota transplantation; OMVs, outer membrane vesicles; SCFA, short‐chain fatty acid; TMAO, trimethylamine N‐oxide; TME, tumor microenvironment.

Together, synergy of microbiome–therapy defines an important class of extrinsic modulation of response. In a tumor context‐specific manner, modulating the hosts’ (patho)ecological baseline involving microorganisms thence can either enhance anticancer therapy by metabolic and immune reprogramming or otherwise contribute to tolerance and toxicity. To outline the primary classes of strategies, types of interventions, and mechanistic bases for microbes’ involvement in and modulation of cancer therapeutics, as well as to compare levels of evidence and relevant clinical scenarios, key studies are compiled in Table 4. Progressive incorporation of microbiome elements into assessment templates for standard care may allow a comprehensive multilevel evaluation of interindividual variation, which can introduce more refined adjustments to therapeutic plans [173]. As illustrated in Figure 7, engineered microbial OMVs and gut‐derived metabolite output can act upstream to potentiate immune‐checkpoint blockade and radio/chemosensitization, thereby providing a mechanistic underpinning for precision nanodelivery and enhancement of standard‐of‐care modalities. Collectively, these strategies highlight the emerging potential of microbiome‐informed interventions in cancer therapy, while emphasizing the need for further mechanistic and clinical validation [10, 11].

**TABLE 4:** Major strategic frameworks and evidence bases for microbiota‐mediated tumor therapy.

Strategy type	Intervention approach	Mechanism of action	Type of evidence	Applicable tumor type/clinical context	Key findings	Reference
Microbial metabolite‐based therapeutic strategy	Butyrate supplementation or ACADS gene knockout	Enhancement of anticancer efficacy through modulation of intracellular calcium homeostasis	Liquid chromatography–mass spectrometry and gas chromatography–mass spectrometry analyses	Hepatocellular carcinoma	Butyrate suppresses hepatocellular carcinoma proliferation and metastasis and enhances the therapeutic efficacy of sorafenib.	[174]
Gut microbial metabolites enhancing cancer immunotherapy	Butyrate treatment	Enhancement of CD8^+^ T‐cell immune responses in an ID2‐dependent manner	Mouse models and human clinical data	Cancer under oxaliplatin chemotherapy	Butyrate enhances CD8^+^ T‐cell responses via ID2, thereby improving oxaliplatin efficacy.	[5]
IL‐17 signaling‐mediated microbial intervention	IL‐17RA deficiency and microbiota ablation	Promotion of tumor growth through IL‐17 signaling and activation of DUOX2 pathways	Mouse and human PDAC models	Pancreatic cancer and brain tumors	IL‐17RA deficiency accelerates tumor growth, while microbiota ablation overcomes resistance to IL‐17RA blockade.	[175]
Gut microbial metabolite‐assisted chemotherapy	Urocanase A (UroA)/UAS03 combined with 5‐fluorouracil (5‐FU)	Reduction of 5‐FU efflux via downregulation of drug transporters	In vitro and in vivo mouse models	CRC with 5‐FU resistance	UroA/UAS03 enhances 5‐FU efficacy and overcomes chemoresistance.	[176]
Traditional Chinese medicine‐assisted microbiota modulation	SSG	Modulation of gut microbiota, improvement of tumor‐associated macrophage polarization, and inhibition of the nuclear factor kappa B (NF‐κB) pathway	Mouse models, fecal microbiota transplantation, and 16S rRNA amplicon sequencing	Pulmonary metastatic cancer	SSG reshapes the gut microbiota, suppresses lung metastasis, and improves tumor‐associated macrophage (TAM) polarization.	[177]
Metabolite‐mediated regulation of cell death	I3A	Activation of AHR, inhibition of JNK/c‐JUN signaling, and stabilization of glutathione peroxidase 4 (GPX4)	Mouse models and mechanistic studies	Melanoma and CRC	I3A suppresses cell death, alleviates oxidative stress, and enhances tumor tolerance.	[178]
Natural compound‐mediated microbiota and signaling modulation	Shikonin and acetylshikonin	Improvement of gut microbiota and regulation of Wnt/β‐catenin signaling	Mouse models and differential proteomics	Colitis‐associated colorectal cancer (CAC)	SK reshapes gut microbiota, suppresses proinflammatory factors, and modulates cancer‐associated signaling pathways.	[179]
Microbiota‐mediated tumor biotherapy	Injection of photosynthetic bacteria combined with visible light irradiation	Hydrogen production induces oxidative stress and mitochondrial apoptosis while activating antitumor immunity	Preclinical studies (in vitro and mouse models)	Hypoxic microenvironments of solid tumors (e.g., BC and melanoma)	Photosynthetic bacteria sustainably produce hydrogen to suppress tumor growth without inducing PD‐L1 upregulation.	[180]
Synbiotic formulation/delivery system	Oral gelatin–inulin encapsulated Lactobacillus capsules	Depletion of glutathione to generate reactive oxygen species (ROS), activation of NLRP3 inflammasome, induction of M1 macrophage polarization, and modulation of short‐chain fatty acid (SCFA)‐producing microbiota	Preclinical studies (animal and in vitro experiments)	Colon cancer/intestinal microenvironment	The hydrogel enhances probiotic colonization and survival, suppresses tumor growth via immune and microbiota modulation, and synergizes with 5‐FU chemotherapy.	[181]
Regulation of gut microbiota and estrogen metabolism	β‐Glucuronidase (GUS) inhibitors	Inhibition of GUS reduces estrogen reabsorption and attenuates tumor progression	Comparative studies between BC patients and healthy women	BC	Alterations in gut microbiota are associated with BC development, and GUS inhibitors exhibit antitumor potential.	[182]
Microbiota‐based immunomodulatory strategy	Oral administration of probiotics combined with intragastric gavage	Inhibition of the TLR4 pathway and reprogramming of M1 macrophage polarization	Preclinical studies (in vitro and animal experiments)	Pancreatic cancer and its tumor microenvironment	Combined probiotic treatment suppresses tumor growth and promotes M1 macrophage polarization.	[183]
Dietary metabolic modulation and immunoprevention strategy	Oral combination of broccoli sprout and Withania somnifera extracts	Upregulation of tumor suppressor and proapoptotic proteins, downregulation of epigenetic enzymes, and remodeling of gut microbiota	Preclinical studies (animal models)	Chemoprevention of triple‐negative BC	The combined intervention significantly reduces tumor incidence and reshapes the gut microbiota.	[184]
FMT‐assisted therapy	Fecal microbiota transplantation	Restoration of gut microbial homeostasis and enhancement of immune checkpoint inhibitor (ICI) efficacy	Clinical applications in ulcerative colitis (UC) and cancer immunotherapy	UC and cancer immunotherapy (e.g., melanoma)	FMT enhances ICI efficacy, although clinical responses remain variable.	[185]
Dietary intervention‐combined therapy	Combined application of wheat bran‐derived component C21 and butyrate	Synergistic induction of apoptosis, autophagy, and endoplasmic reticulum stress in CRC cells	In vitro experiments and mouse model studies	CRC	Combined C21 and butyrate treatment effectively suppresses CRC cell growth and induces apoptosis.	[186]
Microbial metabolite‐mediated regulation	Combined treatment with 2‐methylisocitrate and 5‐FU	Modulation of chemotherapeutic responses via metabolic reprogramming and DNA damage regulation	Cell lines, 3D spheroid cultures, and Drosophila model experiments	Cancer chemotherapy, particularly CRC	2‐Methylisocitrate synergizes with 5‐FU to enhance antitumor efficacy.	[187]
Gut microbiota modulation	Intragastric administration of apple polysaccharides	Regulation of gut microbiota and inhibition of the Wnt signaling pathway	Mouse experiments	CAC	AP reverses gut microbiota dysbiosis and suppresses CAC progression.	[188]
Metabolite‐based therapeutic strategy	Utilization of microbiota‐derived metabolites (e.g., SCFAs)	Immune modulation, apoptosis induction, and remodeling of the tumor microenvironment	Preclinical studies and review analyses	Prevention and treatment of CRC	Microbiota dysbiosis drives CRC, and microbial metabolites possess therapeutic potential.	[189]
FMT‐based combination immunotherapy strategy	Fecal microbiota transplantation combined with anti‐PD‐1 therapy	Reshaping of the gut ecosystem to enhance antitumor immune responses	Phase I clinical trial and immunological analyses	Anti‐PD‐1–refractory metastatic melanoma	FMT improves the immune microenvironment and restores clinical responses in a subset of patients.	[141]
Metabolic intervention and gut microbiota regulation strategy	High‐cholesterol diet combined with cholesterol‐lowering therapy	Hepatocarcinogenesis driven by microbiota and metabolite alterations (e.g., bile acids)	Preclinical animal models and clinical population validation	Prevention of nonalcoholic fatty liver disease‐associated hepatocellular carcinoma	Cholesterol‐lowering therapy prevents liver cancer development and restores microbiota homeostasis.	[57]
Dietary regulation‐induced microbiota‐driven carcinogenesis	High‐fat diet and gut microbiota depletion	Induction of microbiota dysbiosis, metabolic disturbances, and intestinal barrier disruption	CRC mouse models and transplantation experiments	High‐fat diet‐associated colorectal carcinogenesis	High‐fat diet promotes CRC development via microbiota and metabolite alterations.	[190]
Natural compound–microbiota–metabolite axis‐based regulation strategy	Oral administration of ginsenoside Rh4	Enrichment of Akk, promotion of ursodeoxycholic acid (UDCA) production, and activation of farnesoid X receptor (FXR) to suppress the TLR4–NF‐κB pathway	Preclinical studies (animal models combined with microbiota depletion and transplantation)	Prevention and treatment of CRC	Rh4 suppresses CRC in a microbiota‐dependent manner via the Akkermansia–UDCA–FXR axis.	[54]
Immune activation and radiosensitization strategy	Modulation of microbiota or administration of bacterial second messengers	Synergistic activation of the STING pathway by c‐di‐AMP and dsDNA to enhance antitumor immunity	Preclinical studies and validation in clinical patient cohorts	Radiotherapy for hepatocellular carcinoma	Gut microbiota dysbiosis impairs radiotherapy efficacy, with c‐di‐AMP identified as a key regulatory mediator.	[191]
Dietary intervention and metabolic regulation strategy	Ketogenic diet feeding	Enrichment of stearic acid‐producing bacteria, induction of apoptosis by stearic acid, and reduction of Th17 cells	Preclinical studies (animal models and microbiota transplantation validation)	Prevention and treatment of CRC	The ketogenic diet suppresses CRC growth by altering the microbiota and increasing stearic acid levels.	[192]
Natural compound‐mediated gut microbiota anticancer regulation	Berberine intervention targeting high‐fat diet‐associated microbiota	Reshaping of gut microbiota and reduction of lysophosphatidylcholine (LPC) to suppress tumor growth	Mouse models combined with multiomics analyses	High‐fat diet‐associated CRC	Berberine suppresses colorectal carcinogenesis via the microbiota–LPC axis.	[193]
Commensal bacterial vesicle‐mediated immunotherapy modulation	Oral administration of extracellular vesicles derived from Bifidobacterium	Enhancement of anti‐PD‐1 immune responses via the TLR4–NF‐κB pathway	Mouse models and patient‐derived organoids	Immunotherapy for non‐small cell lung cancer	Bacteria‐derived vesicles enhance PD‐1 efficacy and increase CD8^+^ T‐cell infiltration.	[194]

Abbreviations: 5‐FU, 5‐fluorouracil; CAC, colitis‐associated colorectal cancer; FXR, farnesoid X receptor; GPX4, glutathione peroxidase 4; GUS, β‐glucuronidase; ICI, immune checkpoint inhibitor; LPC, lysophosphatidylcholine; NF‐κB, nuclear factor kappa B; ROS, reactive oxygen species; SCFA, short‐chain fatty acid; TAM, tumor‐associated macrophages; UC, ulcerative colitis; UDCA, ursodeoxycholic acid.

## 7 Challenges and Technological Advances in Current Research

### 7.1 Advances in Microbiome Analytics

The fast pace of intratumoral microbiology research is due in part to the ongoing development of microbiome profiling technology, from mere detection to functional annotation. Early culture‐based methodologies were ill‐suited to the low‐biomass, low‐abundance, and fastidious taxa (or organisms in nonconventional metabolic states) that may inhabit tumor tissue, limiting a comprehensive view of intratumoral ecological complexity. The implementation of high‐throughput sequencing has fundamentally reshaped the study of microbiomes, transitioning the field from simple identification to elucidating their impact on tumor biology, underpinned by advanced technical platforms. But increased analytical sensitivity does not prove causality by itself: the hard part is still translating fragile signals in the noisy, contaminated environments of nature to robust mechanistic understanding.

At the community scale, due to mature workflows and affordable cost, 16S rRNA gene sequencing remains widely used in large cohort studies and association analyses; however, it has inherent limitations in taxonomic resolution, functional inference, and the detection of nonbacterial organisms [195]. In contrast, shotgun metagenomics is able to recover total microbial DNA with less taxonomic bias and has facilitated simultaneous reconstruction of community taxonomic profiles, abundance, and variety of functional gene content, and significantly improved resolution of the sequences in intratumoral microbiome investigations. Through metagenomic profiling in a large multisite CRC cohort, these results revealed microbial features associated with tumor anatomical location and supported the development of noninvasive stratification strategies that combine stool profiles with information on tumor location along the colorectum, demonstrating a potential path from community‐scale profiling to clinical application [142]. On top of this, spatially resolved approaches and computational methodologies have extended the dimensionality along which microbe–tumor interactions can be analyzed: through integration of 16S rDNA sequencing, spatial metabolomics, in situ hybridization, high‐resolution imaging, and statistical modeling of large datasets (e.g., from The Cancer Genome Atlas [TCGA]), researchers are able to map microbial distributions while maintaining tissue architecture—and crucially improve their ability to separate biological signal from environmental or reagent‐induced noise. In a few cases, the integration of community profiling with single‐cell transcriptomics, spatial registration, and functional perturbation has started to link community structure to strain‐specific activities and metabolic functions, marking a transition from descriptive association to mechanism‐focused research [146, 196, 197]. However, no single measure of community relationships, spatial colocalization, or multiomics inference can definitively find the difference between functional participation and passive co‐occurrence; rather causal interpretations still depend on experimental design as well as follow‐up intervention studies.

Overall, advances in microbiome analytics have improved detection, resolution, and interpretability, extending intratumoral studies from presence/absence to spatial context and functional hypotheses. However, rising technical resolution has not resolved the causal bottleneck. Whether derived from metagenomic associations or from single‐cell and spatial colocalization signals, conclusions remain highly sensitive to analytical pipelines and contamination‐control strategies. Computational integration and multiomics modeling deepen inference, but also amplify interpretive complexity—reinforcing a persistent tension between expanding technical capability and the need for disciplined biological claims.

### 7.2 Challenges in Data Integration and Bioinformatics

As studies of the intratumor microbiome transition from correlative cataloging to mechanistic dissection and clinical translation, data integration and bioinformatic inference constitute a central methodological bottleneck. Contrary to traditional microbiome studies, the tissues of tumors are typically low‐biomass environments and dominated by host DNA, which not only increases technical noise but also makes it difficult to capture true microbial signals that need to be comparable across studies and biologically interpretable. Under this noise‐rich condition, robust and replicable analyses are crucial for the field to move from “detectable” to “explainable.”

At the data level, separating true microbial signal from contamination is still the most disputed—and critical—analytical issue. Contaminants derived from reagents, interlaboratory variation and batch effects may overwhelm the outputs of a sequencing experiment, while heterogeneity in contamination control, filter thresholds, and reference databases often results in discordant conclusions including different directions of association within the same tumor type. This instability does not only affect the credibility of the microbial abundance but also impacts functional inference and mechanistic assertions downstream. Large‐scale cancer cohorts and statistical modeling have been used to denoise the biological signals from systematic noise, thus providing a more standardized tumor‐associated microbial atlas; high‐quality, well‐powered cohorts may partially prevent the issue of statistical fragility—for instance, such approach showed that a relatively conserved set of core pathobionts and virulence determinants can be identified in CRC despite strong interindividual heterogeneity [198, 199]. However, these methods do not completely remove the systematic bias due to technical variation and nonharmonized analytical pipelines.

Single‐omics level robustness is also too weak to solve the problem of microbial function inside tumors, so there is no way around multiomics integration. Existing studies are being developed to increasingly be able to integrate 16S rRNA profiling, metagenomics, metabolomics, spatial‐omics, and clinical phenotypes together but the differential scale, resolution, and noise structure in these modalities collectively form a technical barrier for cross‐layer inference. While machine learning and high‐dimensional modeling have been extensively applied to locking the patterns associated with tumor phenotypes or treatment response, these outputs are often purely correlative, have limited biological interpretability, and cross‐cohort generalizability, reflecting a continued trade‐off between prediction parsimony and mechanistic insight [200]. These limitations are particularly evident when integrating across sample types and spatial hierarchies—such as joint microbiome and spatial metabolomics to interrogate the gut–blood–tumor axis, or metagenome–metabolome integration in intervention studies—where workflow standardization and reproducibility remain substantial hurdles [196, 201].

In general, data integration and bioinformatic analysis are the main methodological bottleneck in the field of intratumoral microbiome research. While low microbial biomass and abundant host background exaggerate technical artefacts, computational discrimination of true signals from contamination is challenging, exacerbating cross‐study inconsistency. Although large cohorts and models favor stability, there is a systemic bias. Multiomics techniques hold the key to functional insight, but are limited by modal discordance and interpretability, with gains in prediction not always translating into increased mechanistic understanding. Balancing robustness, complexity and interpretability will be crucial to turning associations into trustworthy mechanistic conclusions.

### 7.3 Sample Handling and Experimental Design Considerations

As the field of intratumor microbiome shifts from descriptive observations to mechanistic dissection and clinical translation, precise sample processing and study design form the methodologic foundation for causal inference. In comparison with gut or environmental microbiology, tumor tissues consistently contain extremely low microbial biomass embedded within an excess of host DNA such that even mild preanalytical variation, batch effects, or sub‐optimal design decisions can be disproportionately inflated and confuse the assessment of the authenticity of a signal. Therefore, in this field, the sample‐level systematic manipulation is considered as the most basic and challenging requirement.

Harvesting and processing at the sampling location, workflow in operation, as well as storage conditions may be crucial. Tumors are characterized by spatial heterogeneity in hypoxia, immune infiltration, and metabolic state, as are microbial localized distributions. Without a defined set of regions to sample from and accounting for the heterogeneity in tissue, locally overrepresented signals might be misinterpreted as tumor features. Furthermore, low‐biomass samples are naturally prone to contamination: introduced extrinsic microbes during sample sectioning, as well as during DNA and library preparation processes, can overshadow their subsequent profiles. Inclusion of negative controls, process blanks, and technical replicates from the beginning—and treating surveillance for contamination as an embedded design feature—remains a necessity for credibility.

Similarly, adequate controls and staged validation pathway are key to avoid systematic bias. Accumulating evidence suggests that there is more to pathogenicity or regulation than mere correlation, and multifaceted “discovery–validation–causal verification” paradigm‐type frameworks should be applied. For instance in the bacterium–metabolite‐centered hypotheses, researchers have utilized a series of techniques from metagenomic screening in clinical samples, bacterial isolation, and metabolite identification through to functional testing either in vitro or in vivo together with molecular docking and finally genetic manipulation to develop a consistent microbe–host pathogenic chain [74, 202]. Extending this logic, integration of multicenter, large‐cohort tissue, and fecal datasets with explicit stratification by spatial source has enabled cross‐sample‐type and multiomics validation under clinically relevant conditions, directly probing microbial contributions to tumor initiation and progression, thereby strengthening causal inference [202, 203, 204, 205]. Collectively, these studies underscore the value of coordinated design linking clinical materials, functional models, and multiomics readouts.

**FIGURE 8:**
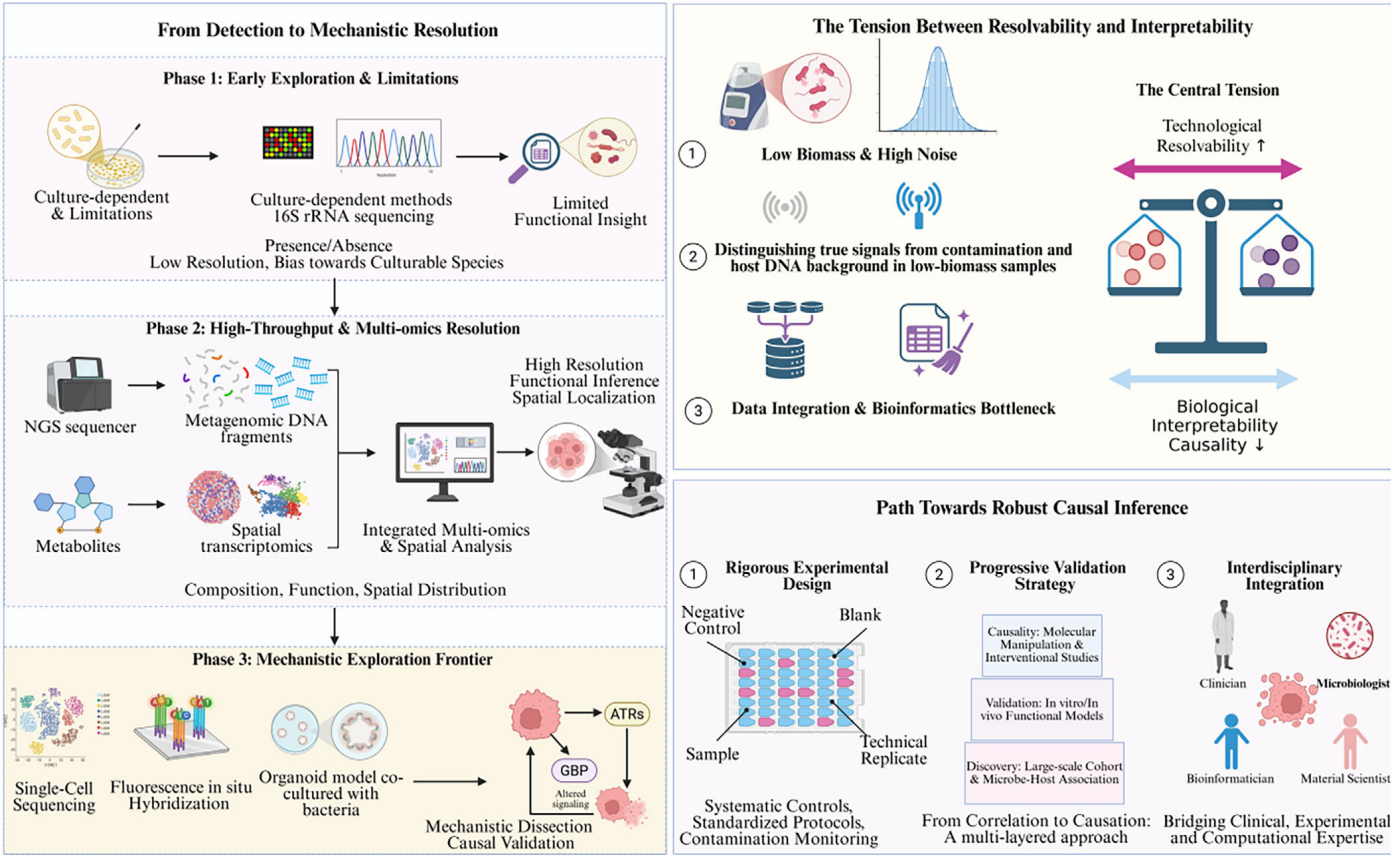
Methodological challenges and emerging technologies in intratumoral microbiome research. In‐depth intratumoral microbiome study is limited by a major methodological bottleneck: the analytical compromise between accurate mechanistic attribution and resolution. Early methods used culture‐based approaches and 16S rRNA amplicon sequencing, with dependencies on low‐biomass samples and culturability bias resulting in a high noise‐to‐signal ratio, poor signal resolution, and shallow functional interpretation. High‐throughput phases have utilized next‐generation sequencing (NGS) for shotgun capture of metagenomic DNA fragments, while integration with metabolomics and spatial transcriptomics enables multiomic dissection of community composition, functional inference, and physical organization; however, cross‐modality scale discrepancies and differences in noise structures challenge integrative bioinformatics and model interpretability. At the mechanistic front, single‐cell sequencing and in situ fluorescence in situ hybridization (FISH) enable causal deconvolution and functional validation of strain–host interactions, as well as directed interrogation of pathways connecting microbial metabolic stress with host DNA repair, including oxidative repair proteins such as Nei‐like DNA glycosylase 2 (NEIL2) and nonhomologous end joining (NHEJ) pathways. In conclusion, the tractability of intratumoral microbial signals and their translational potential increase with emerging technologies, but robust causal inference will depend on standardized control frameworks, multicenter validation, and highly interpretable models (by BioRender). FISH, fluorescence in situ hybridization; NEIL2, Nei‐like DNA glycosylase 2; NGS, next‐generation sequencing; NHEJ, nonhomologous end joining.

However, practice reveals continuing structural contradictions. Given such ultra‐low biomass and profound spatial heterogeneity, sampling position, handling and storage have outsize effects on focal detection, yet there is seldom an obvious threshold at which one might infer that such detection is applicable to tumor‐level biology. While bolstering confidence, these staged designs and multilayered functional validation efforts impose significant constraints on cost, specimen availability, and reproducibility, thereby limiting scalability. This gap between methodological ideals and real‐world conditions leaves parts of the literature anchored at the level of association, highlighting a practical bottleneck in translating sample‐level signals into robust causal attribution. As highlighted in Figure 8, emerging multiomics and in situ functional models are improving both interpretability and spatial precision of intratumor microbial signals; however, durable causal claims will still depend on stringent control architectures coupled with independent, cross‐center replication.

## 8 Discussion and Outlook

Recent studies on the intratumoral microbiota have expanded the understanding of TME complexity. Tumors are coming to be understood less as a simple binary of malignant cells and host immunity, and more as multilayered, dynamic ecosystems that additionally include microbial components. The available evidence indicates that, in general terms, intratumoral microbes do not act in isolation: their activities are integrated into the systemic immune and metabolic context sculpted by tumor‐associated organisms from the gut or other nearby sites. Instead of just bystanders, these communities may represent functionally integrated components during key steps in tumorigenesis, progression, and therapeutic response through sustained immunomodulation, metabolic rerouting, maintenance of inflammatory signals, and integration into cellular communication systems. These influences are profoundly context dependent, and the sign and magnitude of their impact is determined by tumor type, microbial composition, host immune state, and treatment history [206]. Apparent discrepancies across studies therefore need not indicate irreproducibility, but may instead expose the intrinsic heterogeneity and dynamic plasticity of tumor–microbe interactions.

From a mechanistic perspective, the biology mediated by intratumoral microbes is resistant to simplification into a single pathway or main effect. Rather, TME function is fine‐tuned by systemic regulation across immune, metabolic, inflammatory, and signaling axes. Immunologically, microbial antigenic stimuli can reprogram the equilibrium between activation and suppression by modifying innate immune differentiation, efficiency of antigen presentation, and proficiency of effector T cells [207, 208]. Metabolically, microbially mediated disruptions in carbon utilization and amino‐acid, lipid, and bile‐acid metabolism converge with immunometabolic crosstalk, modulating tumor development and immune evasion. On the genetic and signaling front, chronic inflammation associated with microbe exposure, DNA damage, and rewiring of key signaling nodes may contribute to a substrate for adaptive tumor evolution. These dimensions are not independent but rather constitute mutually reinforcing circuits of regulation, emphasizing the necessity of transitioning from single‐factor to system‐level mechanistic integration [209].

Though this is in rapid development, methodological limitations still hinder the robustness and comparability of these. The contamination risk endemic to low‐biomass samples, cross‐platform, and analytical‐pipeline variability, and scale mismatching during the integration of multiomics introduce considerable uncertainty in signal ascription and functional interpretation [210]. Furthermore, many studies are still correlation‐driven without staged cross‐level causal validation, limiting the depth and confidence in mechanism [211]. To address this limitation, future studies should increasingly incorporate functional validation frameworks, including gnotobiotic or germ‐free animal models, controlled microbial perturbation experiments, and causal intervention strategies [212]. In addition, emerging approaches such as CRISPR‐based microbial engineering and strain‐level manipulation provide opportunities to directly interrogate microbe–host interactions and establish mechanistic links between specific taxa and tumor phenotypes [213, 214]. Future studies integrating mechanistic validation with well‐designed clinical evidence will be essential to distinguish causal effects from correlative associations and to define which findings are generalizable across tumor types. It is anticipated that future progress will require more stringent standardization in terms of sample handling, control design, data processing, and functional validation; multicenter large‐cohort studies; and the integration of spatial and single‐cell technologies, to bridge the gap between mere detection of microbes and determining their biological relevance.

In the future, precision oncology will be the biggest impact of this field. Microbiome‐informed diagnostics, prognostics, and therapy‐response models could have synergy with existing molecular biomarkers to further stratify tumors and for risk assessment. Concurrently, microbiota‐targeted or microbiota‐enabled interventions, from immunometabolic reshaping using tumor‐associated microbes to targeted delivery systems built for the local TME, provide potential avenues to breach current therapeutic plateaus [215, 216]. However, clinical translation will depend on systematic evaluation of safety, controllability, and interindividual variability. Only by anchoring research in a mechanistically grounded, clinically driven framework can intratumoral microbiota move from an emerging focus to a tractable layer of therapeutic control, providing sustained momentum for precision cancer medicine.

## Author Contributions

Haoling Zhang, Zengkan Du, Ping Lu, and Aimin Jiang contributed to the manuscript writing and figure preparation. Zhijing Song, Bing Dai, and Wangzheqi Zhang designed the work. Yadong Guo and Yawei Liu supervised the work. All authors read and approved the final manuscript.

## Ethics Statement

The authors have nothing to report.

## Conflicts of Interest

The authors declare no conflicts of interest.

## Funding

This work was supported by grants from the National Natural Science Foundation of China: 81970640 and 82370735; Gansu Province Science and Technology Plan Project ‐ Key R&D Program‐International Science and Technology Cooperation Category: 23YFWA0005; Gansu Provincial University Innovation Fund: 2025A 110; Northwest China Center for Collaborative Innovation in TCM Prevention and Treatment of Nutrition and Environment‐Related Diseases: ZYXT ‐ 24 02; Department of Science and Technology of Gansu Province, Natural Science Foundation: 22JR5RA582: 2023A 088.

## Data Availability

Data sharing is not applicable for this article as no datasets were generated or analyzed during the current study. All information is derived from publicly available articles and datasets.
